# High-Performance Perovskite Quantum Dot Solar Cells Enabled by Incorporation with Dimensionally Engineered Organic Semiconductor

**DOI:** 10.1007/s40820-022-00946-x

**Published:** 2022-10-17

**Authors:** Seyeong Lim, Dae Hwan Lee, Hyuntae Choi, Yelim Choi, Dong Geon Lee, Sung Beom Cho, Seonkyung Ko, Jongmin Choi, Younghoon Kim, Taiho Park

**Affiliations:** 1 Department of Chemical Engineering, Pohang University of Science and Technology (POSTECH), Pohang, 37673 Republic of Korea; 2 Department of Materials Science and Engineering, Hanyang University, Seoul, 04763 Republic of Korea; 3 Center of Materials Digitalization, Korea Institute of Ceramic Engineering and Technology (KICET), Jinju, 52851 Republic of Korea; 4 Department of Materials Science and Engineering, Ajou University, Suwon, 16499 Republic of Korea; 5 Department of Energy Science and Engineering, Daegu Gyeongbuk Institute of Science and Technology (DGIST), Daegu, 42988 Republic of Korea; 6 Department of Chemistry, Kookmin University, Seoul, 02707 Republic of Korea

**Keywords:** CsPbI_3_ quantum dots, Star-shaped organic semiconductors, Hybrid perovskite quantum dots, Solar cell stability, High-efficiency photovoltaics

## Abstract

Perovskite quantum dots (PQDs) have been considered promising and effective photovoltaic absorber due to their superior optoelectronic properties and inherent material merits combining perovskites and QDs. However, they exhibit low moisture stability at room humidity (20–30%) owing to many surface defect sites generated by inefficient ligand exchange process. These surface traps must be re-passivated to improve both charge transport ability and moisture stability. To address this issue, PQD-organic semiconductor hybrid solar cells with suitable electrical properties and functional groups might dramatically improve the charge extraction and defect passivation. Conventional organic semiconductors are typically low-dimensional (1D and 2D) and prone to excessive self-aggregation, which limits chemical interaction with PQDs. In this work, we designed a new 3D star-shaped semiconducting material (Star-TrCN) to enhance the compatibility with PQDs. The robust bonding with Star-TrCN and PQDs is demonstrated by theoretical modeling and experimental validation. The Star-TrCN-PQD hybrid films show improved cubic-phase stability of CsPbI_3_-PQDs via reduced surface trap states and suppressed moisture penetration. As a result, the resultant devices not only achieve remarkable device stability over 1000 h at 20–30% relative humidity, but also boost power conversion efficiency up to 16.0% via forming a cascade energy band structure.

**Supplementary Information:**

The online version contains supplementary material available at 10.1007/s40820-022-00946-x.

## Highlights

- We designed and developed a 3D star-shaped conjugated molecule (Star-TrCN) to incorporate organic semiconductor with CsPbI_3_ perovskite quantum dots (PQDs).
- The robust chemical bonding of Star-TrCN and PQDs is demonstrated by theoretical modeling and experimental validation, which significantly improved the cubic-phase stability of CsPbI_3_-PQDs by passivating vacant sites and preventing moisture penetration.
- The Star-PQD hybrid solar cells not only achieved remarkable device stability over 1000 h at 20–30% relative humidity, but also boosted power conversion efficiency up to 16.0% via cascade energy band structure.

**Supplementary Information:**

The online version contains supplementary material available at 10.1007/s40820-022-00946-x.

## Graphical abstract

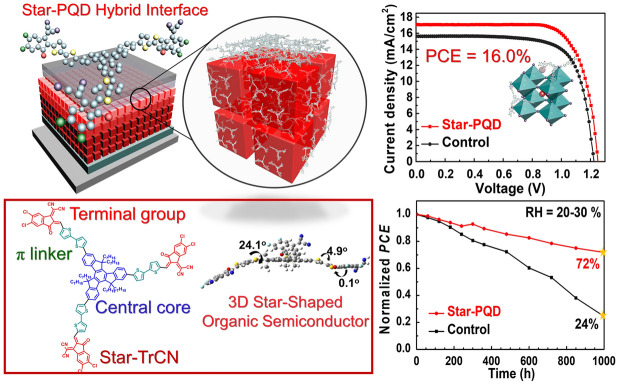

## Introduction

Organic–inorganic hybrid perovskite solar cells (PSCs) have shown remarkable device performance. Through optimizing the compositional alloying, post-processing step, interfacial engineering and device architecture [1–5], researchers have increased the power conversion efficiency (PCE) of PSCs to above 25% [6]. However, the long-term stability of PSCs is limited by degradation of the perovskite absorbers [7, 8]. Under operational circumstances (i.e., high temperature or humidity), hybrid perovskites decompose into PbI_2_. Moreover, the organic compounds used as the monovalent cations of the perovskite structure (ABX_3_) (e.g., methylammonium, ethylammonium and formamidinium) are volatile, reducing the device stability [9].

Recently, replacing the organic cation (the A site of ABX_3_) with an inorganic cation has been regarded as a facile strategy for improving the stability of PSCs [10–12]. Among these all-inorganic PSCs, CsPbI_3_ is a most promising photovoltaic absorber because its bandgap (*E*_g_ = 1.73 eV) is appropriate for the single-junction solar cell applications [13]. However, the photoactive cubic-phase CsPbI_3_ (*α*-CsPbI_3_) tends to transform into the more thermodynamically stable non-photoactive phase (*δ*-CsPbI_3_) below 320 °C [14].

Luther and co-workers proposed a perovskite quantum dot (PQD) strategy to overcome the cubic-phase instability issues of bulk CsPbI_3_ [15]. The cubic-phase stability of CsPbI_3_ is much higher in PQDs than in bulk perovskites because the surface strain increases according to the decreases in the crystal size of perovskites with surface ligand stabilization [16]. Moreover, CsPbI_3_-PQDs exhibit superior optical and electronic properties such as size-tunable *E*_g_, high photoluminescence quantum yield, high absorption coefficient and high charge carrier mobility [17, 18]. Consequently, the CsPbI_3_-PQDs have been considered promising and effective photovoltaic absorbers [19, 20].

Although CsPbI_3_-PQDs exhibit outstanding photovoltaic properties, their moisture stability should be further improved. In the PQD synthetic process, the PQDs have been stabilized with long-chain hydrocarbon ligands such as oleic acid and oleylamine (OLA) [21, 22]. Native ligands enhance the colloidal stability of PQDs [13, 23, 24], but their insulating properties prevent efficient charge transport. To enhance the electronic coupling within PQDs, the long-chain ligands should be replaced with short ligands (e.g., acetate) through the solid-state ligand exchange process [13, 25]. Although the ligand exchange process increases the charge transport ability in PQD films [26, 27], the inefficient ligand exchange can induce surface trap states of the PQDs [28]. Moreover, under the device operating conditions, moisture can penetrate the PQDs through the surface defect sites, causing the PQD damage, low device performance and stability [29]. The most widely used hole transport material (HTM) in high-efficiency PQD solar cells is a spiro-OMeTAD, which requires dopants [e.g., lithium salt (Li-TFSI) and cobalt complexes (FK209)] that easily absorb moisture and accelerate the degradation of the device stability [30, 31].

To improve the moisture stability of PQDs, several researchers have attempted to combine PQDs with organic conjugated molecules, forming hybrid PQDs (HPQDs) [29, 32–34]. The organic conjugated molecules not only provide a physical hydrophobic barrier to prevent moisture penetration of HPQDs, but also passivate the surface defects through their various functional groups (e.g., –COO, –CN and halide) [35]. Ma et al. [33] and Hu et al. [36] reported that the charge collection efficiency in PQD devices can be significantly enhanced with hybrid configurations (e.g., HTM and hetero-interface architectures), highlighting the advantages of HPQDs. Even though typical low-dimensional [i.e., one- and two-dimensional (1D and 2D, respectively)] organic semiconductors (e.g., polymers and fused-ring small molecules) show high efficiency in organic-based optoelectronics owing to their excellent crystalline properties, charge carrier extraction from the PQDs to the organic semiconductors is inefficient for the HPQD system due to the large difference in their surface energies [17]. Further, the low-dimensional organic semiconductors are prone to excessive self-aggregation, resulting in poor interaction with PQDs [37]. Therefore, a new organic semiconductor design is required to enhance the compatibility between organic semiconductors and PQDs.

Here, considering the dimensionality in engineering organic semiconductors, a newly designed and synthesized three-dimensional (3D) star-shaped semiconductor (Star-TrCN) is combined with CsPbI_3_-PQDs to develop a successful HPQD system. The distinctive structure of star-shaped semiconductors could provide tunable energy levels, high charge carrier mobility and isotropic charge transfer abilities in contrast to 1D semiconductors. Therefore, the Star-TrCN is employed as the interlayer between the HTM and PQDs, and is expected to simultaneously improve the device performance and stability of PQD solar cells. This core structure [10,15-dihydro-5H-diindeno[1,2-a;1ʹ,2ʹ-c] fluorene (truxene)] is facilely synthesized and undergoes high π-conjugation with fluorene, thus facilitating charge transport [38–40]. Star-TrCN is designed by adjusting the energy level between the PQD and HTM layers in density functional theory (DFT) calculations. Truxene with a fused-ring aromatic structure confers hydrophobic properties to the upper layer of PQDs, preventing moisture penetration. Furthermore, the twisted structure of 3D star-shaped Star-TrCN more significantly suppresses self-aggregation than the linear structures of 1D molecule, increasing the compatibility between organic semiconductors and PQDs. Owing to these unique characteristics, 3D star-shaped structures have been discussed as efficient passivator of perovskite surface defects, improving the operational device performance in multiple ways [2, 41–43]. Star-TrCN also possesses several functional groups (–CO, –Cl and –CN) that can passivate the surface defects and result in chemical bonding with PQDs. The Star-PQD hybrid film significantly enhances the cubic-phase stability by passivating the surface defects and preventing moisture penetration through the hygroscopic metal complex dopants in HTM (Spiro-OMeTAD). In long-term stability tests, Star-PQD-based solar cells retained 72% of their initial PCE at ambient conditions (20–30% RH) even after 1000 h. The cascade energy level structure further improves the charge extraction, achieving 16.0% PCE. The 3D star-shaped organic semiconductor design promises the simultaneous improvement of the stability and efficiency of HPQD-based solar cells.

## Experimental

### Materials

All the organic chemicals and solvents were purchased from Sigma-Aldrich and Alfa Aesar and used directly without further purification. Lead iodide (PbI_2_), 1-octadecene (ODE), oleic acid (OA), cesium carbonate (Cs_2_CO_3_), n-hexane, n-octane and lithium bis(trifluoromethylsulfonyl)imide (Li-TFSI) were purchased from Alfa Aesar. Oleylamine (OLA), sodium acetate (NaOAc), chlorobenzene (CB), chloroform (CF), acetonitrile (AN), methyl acetate (MeOAc) and ethyl acetate (EtOAc) were purchased from Sigma-Aldrich. Phenethylammonium iodide (PEAI) was purchased from GreatcellSolar. TiO_2_ precursor solution (SC-BT060) and 2 M TiCl_4_ aqueous solution were purchased from Sharechem. 2,20,7,70-Tetrakis(N,N-di-p-methoxyphenylamine)-9,90-spirobifluorene (Spiro-OMeTAD) was purchased from Lumtec. 2-Amylpyridine was purchased from TCI.

### Synthesis of Cs-oleate Solution

Cs_2_CO_3_ (0.407 g), ODE (20 mL) and OA (1.25 mL) were added to a 250-mL three-necked flask. The system was vacuumed at 120 °C for 0.5 h under stirring. Then, N_2_ was introduced into the system and the solution was stored at this condition before injection.

### Synthesis of CsPbI_3_-PQDs

PbI_2_ (0.5 g) and ODE (25 mL) were added to a 100-mL three-necked flask. The system was vacuumed at 120 °C for 30 min. Then, OA (2.5 mL) and OLA (2.5 mL) were added to the system, and the system was vacuumed at 120 °C for 0.5 h. Thereafter, N_2_ was introduced into the system and heated to 180 °C. At 180 °C, Cs-oleate (2 mL) was quickly injected to the system, and the system was cooled after 10 s to room temperature under ice-water bath.

### Purification of CsPbI_3_-PQDs

CsPbI_3_-PQD solution was purified through two steps. In the first step, MeOAc (30 mL) was added to 15 mL of CsPbI_3_-PQD crude solution. The system was centrifuged at 5 krpm for 3 min. Then, the as-prepared CsPbI_3_-PQD precipitate was dispersed n-hexane (5 mL). In the second step, MeOAc (7 mL) was added to the system. The system was centrifuged at 5000 rpm for 3 min. Then, as-prepared CsPbI_3_-PQD precipitate was dispersed n-hexane (15 mL). The solution was centrifuged at 5 krpm for 3 min. Thereafter, the purified CsPbI_3_-PQD solution was collected. Finally, this solution was dried under vacuum, and the achieved CsPbI_3_-PQD solid was dispersed in n-octane (75 mg mL^−1^). The size of the synthesized PQDs was approximately 10–12 nm (Fig. S1).

### Fabrication of CsPbI_3_-PQD Solar Cells

The c-TiO_2_ electron transport layer was spin-coated on the FTO substrates at 3 krpm for 0.5 min, and the substrates were annealed at 500 °C for 1 h. After the substrates cooled to room temperature, the substrates were immersed in TiCl_4_ aqueous solution (120 mM) at 70 °C for 1 h. Then, they were washed by deionized water and annealed at 500 °C for 1 h. The CsPbI_3_-PQD solution (75 mg mL^−1^ in n-octane) was spin-coated onto the c-TiO_2_ deposited substrates at 1 krpm for 20 s followed by 2 krpm for 5 s. The CsPbI_3_-PQD film was immersed in ligand solution of NaOAc in MeOAc (1 mg mL^−1^) and spin-dried. Thereafter, the ligand-exchanged CsPbI_3_-PQD film was washed by MeOAc for washing. The step was repeated for 5 times to achieve optimum thickness (~ 250 nm). Subsequently, thick CsPbI_3_-PQD film was immersed in ligand solution of PEAI in EtOAc (1 mg mL^−1^) and spin-dried. After that, for Star-TrCN treatment, thick CsPbI_3_-PQD film was soaked in Star-TrCN solution in CF (1 mg mL^−1^) for 5 s and spin-dried. The spiro-OMeTAD hole transport layer was prepared by mixing spiro-OMeTAD (72.3 mg), CB (1 mL), 2-amylpyridine (28.7 μL) and 17.6 μL of Li-TFSI solution in AN (520 mg mL^−1^). This solution was spin-coated on the thick CsPbI_3_-PQD film at 4 krpm for 30 s. Finally, top electrodes (MoO_x_ and Ag with thicknesses of 15 and 120 nm) were deposited by using thermal evaporation equipment.

### Characterization

Cyclic voltammetry (CV) analyses were carried out witha CHI 600C potentiostat (CH Instruments) at a scan rate of 100 mV s^−1^ in 0.1 M solution of tetrabutylammonium perchlorate in acetonitrile. Cross-sectional scanning electron microscopy (SEM) image of CsPbI_3_-PQD solar cell was acquired by using a Hitachi SU8230 equipment. UV–Vis absorption spectra of CsPbI_3_-PQD films were measured by employing a Shimadzu UV-2600 spectrophotometer. X-ray photoelectron spectra (XPS) spectra were measured by using a Thermo Scientific ESCALAB 250Xi analyzer. For preparing grazing-incidence small-angle X-ray scattering (GIWAXS) samples, 1 by 1 cm^2^ silicon wafers were used as substrates. Solutions (Star-TrCN, ITIC, Y6, PBDB-T, CsPbI_3_-PQD and Star-PQD) were spin-coated on the substrates. The GIWAXS analysis was conducted at the Pohang Accelerator Laboratory (beamline 9A, Republic of Korea) with incidence angles between 0.1 and 0.3°. X-ray diffraction (XRD) patterns were measured by using a Bruker D2 Phaser diffractometer. Photoluminescence (PL) spectra of CsPbI_3_-PQD films were measured by employing a Horiba Scientific Flouromax-4 spectrophotometer. Time-resolved PL (TRPL) decay curves of CsPbI_3_-PQD films were measured by employing a Hamamatsu Quantaurus-Tau C11367 spectrometer. The *J* − *V* curves, light intensity-dependent *V*_OC_ and stable power output were acquired by using a Newport Oriel Sol 3A solar simulator equipped with a 450 W Xe lamp and a Keithley 2400 sourcemeter under a simulated air-mass 1.5 global spectrum at 100 mW cm^−2^. External quantum efficiency (EQE) spectra of CsPbI_3_-PQD solar cells were measured by employing a Newport Oriel QuantX-300 incident photon-to-current efficiency (IPCE) equipment with an Oriel Cornerstrone 130 monochromator. DFT calculations with the B3LYP/6-31G (d, p) basis set were performed utilizing Gaussian 09 to evaluate the molecular conformations and electrostatic potential. In particular, alkyl chains were replaced with a methyl or isobutyl group to simplify the calculations and reduce the computational time. Depth profiling of PQD solar cells was analyzed by time-of-flight secondary ion mass spectrometry (ToF-SIMS) using ToF-SIMS IMS 6F equipped with Cs^+^ Gun (impact energy: 5 keV, Current: 5 nA, Raster size: 300 ×  μm^2^ and Analysis Area: 60 um(Φ)). Atom force microscopy (AFM) images of CsPbI_3_-PQD films were measured by employing a Park System NX10 microscope.

## Results and Discussion

### Design Strategy of Star-TrCN Incorporated CsPbI_3_-PQD Solar Cells

**Fig. 1:**
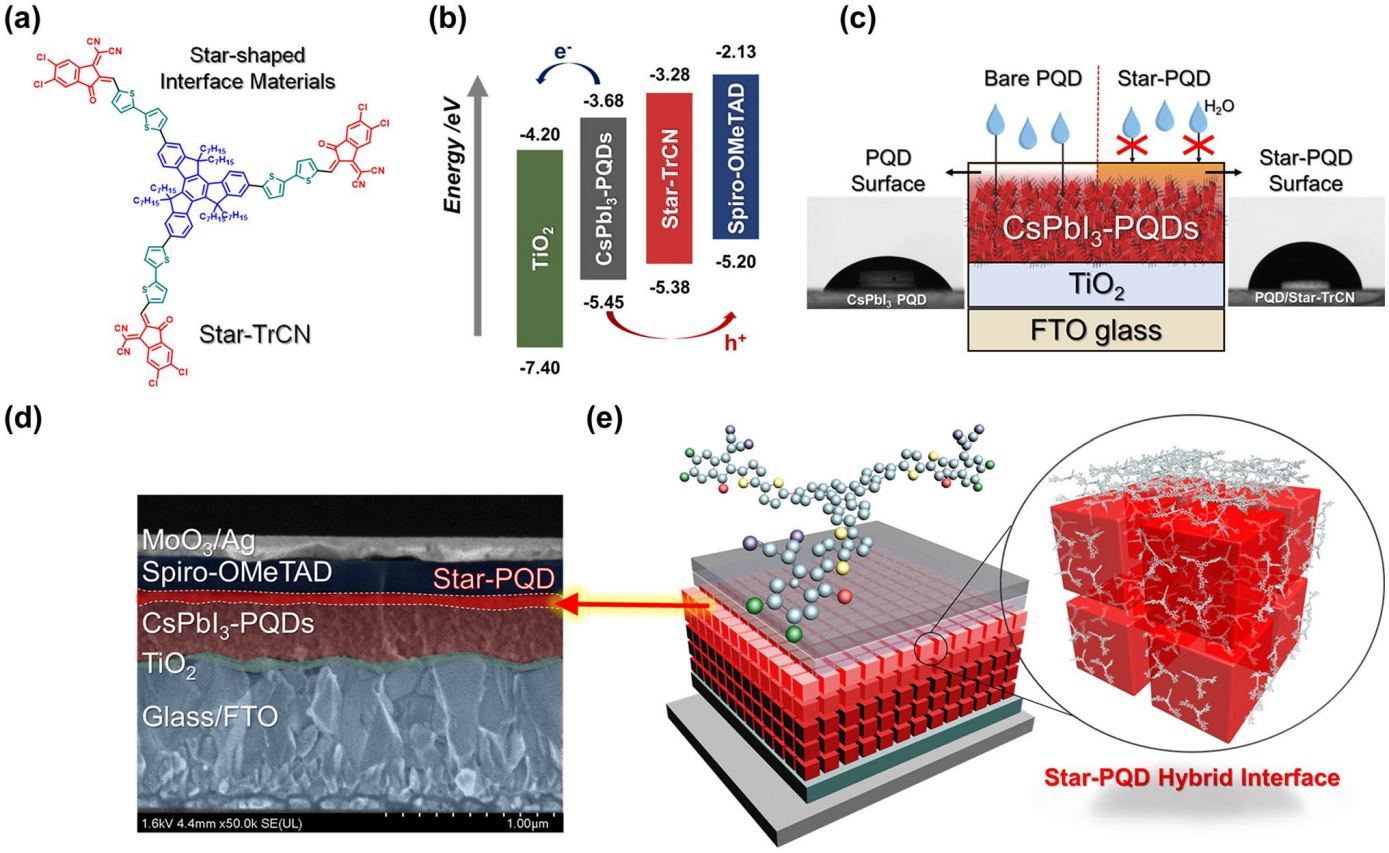
**a** Chemical structure of Star-TrCN. **b** Energy level diagram of charge transfer between CsPbI_3_-PQDs and each transport layer. **c** Schematic showing moisture penetration impedance of the Star-PQD formation. **d** Cross-sectional SEM image of the fabricated device and the device architecture with Star-PQD hybrid interfaces. **e** Schematic of the architecture of PQD solar cells with a Star-PQD hybrid interface

The chemical structure and synthetic routes of Star-TrCN are shown in Fig. 1a and Scheme S1. The detailed synthetic procedures and characterizations are provided in the supporting information. The synthesis of the star-shaped semiconductors targets a truxene core with three fluorine units sharing a central benzene ring. To obtain an adequate energy level and modulate the twisted 3D structure, truxene was expanded by a linker with bi-thiophene through the Suzuki cross-coupling reaction and attached to the terminal group via Knoevenagel condensation. The chemical structures of the intermediates and final molecules were confirmed by ^1^H-, ^13^C-NMR, matrix-assisted laser desorption time-of-flight mass (MALDI-TOF-MS) spectrometry and an elemental analysis (EA) (Figs. S2–S4). During the synthesis, the core, linker and terminal group of Star-TrCN were finely controlled to modulate the energy levels of HTM and the PQDs. The resulting Star-TrCN was characterized by cyclic voltammetry and ultraviolet–visible (UV–Vis) absorption spectroscopy (Fig. S5). The highest occupied and lowest unoccupied molecular orbital energy levels of Star-TrCN were calculated to be − 5.38 and − 3.28 eV, respectively. The cascade formation using Star-TrCN for interfacial energy level alignment improved the charge extraction between PQDs and HTM (Fig. 1b). Moreover, their multiple aromatic ring structures provide superior hydrophobic properties to the PQDs (Fig. 1c). The excellent hydrophobicity of these structures effectively prevents moisture penetration. Figure 1d shows a schematic and a cross-sectional SEM image of the CsPbI_3_-PQD solar cells. The device was fabricated through solid-state ligand exchange of CsPbI_3_-PQDs, which preserves the original crystal size of the PQDs, onto the electron-transporting material (compact TiO_2_). After sequentially depositing the PQDs, the Star-TrCN interface layer was spin-coated on the top PQD surfaces to form Star-PQD hybrid films. Finally, spiro-OMeTAD was spin-coated as an HTM, followed by sequential deposition of MoO_*x*_ and Ag (see the Experimental Section for details) (Fig. 1e).

The Star-TrCN is dissolved in volatile organic solvents [e.g., chloroform (CF), chlorobenzene (CB)] and, as shown in the experimental section, we dissolved Star-TrCN in CF and spin-coated onto the PQD films to form the Star-PQD layer. However, the spiro-OMeTAD is also dissolved in the same organic solvents, which can damage on Star-TrCN layer during spiro-OMeTAD deposition. Although the Star-TrCN layer could be flushed away after spiro-OMeTAD deposition, there is a solvent resistance to CB because the Star-TrCN penetrates into the PQD layer and can strongly result in the chemical bonding with the PQD. We confirmed the robust solvent resistance of Star-PQD film using UV–Vis and nanoscale Fourier transform infrared (nano-FTIR) spectroscopy measurements. When the PQD film was treated with Star-TrCN, the proportion of Star-TrCN absorption range (i.e., 400–550 nm, Fig. S5c) was increased from that of bare PQD. Next, the Star-PQD films were flushed with CB solvent as done in the HTM spin coating process. The flushing process did not significantly affect the absorbance spectrum. In addition, we also performed nano-FTIR spectroscopy measurement. The nano-FTIR can visually show the presence of Star-TrCN depending on the intensity of its functional group [44]. As shown Fig. S6, the FTIR spectrum of the Star-TrCN showed a peak at 1500–1700 cm^−1^, which is related to the stretching of C–C bonds in the aromatic ring of Star-TrCN. In the amplitude image, reflecting the topological information, we can observe the distribution of Star-TrCN. Similarly, the phase image also indicated the wide distribution of Star-TrCN, which shows that Star-TrCN was well incorporated into the PQDs as an interfacial material following the HTM deposition process (CB solvent).

### Chemical Interaction Between Star-TrCN and CsPbI_3_-PQDs

**Fig. 2:**
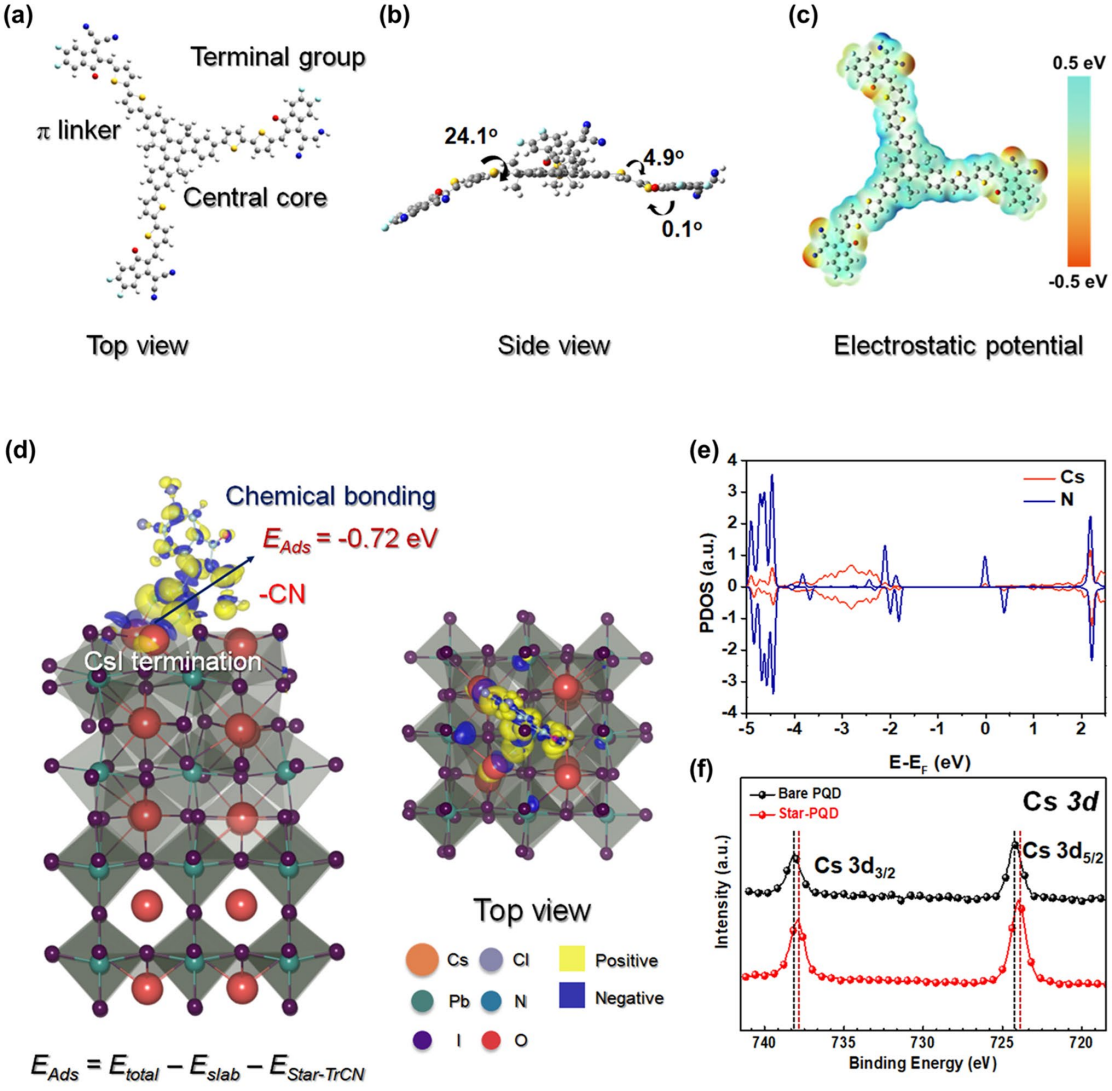
**a** Optimized geometries, **b** dihedral angles and **c** electrostatic potential of the Star-TrCN using DFT calculation at B3LYP/6-31G (d, p) level. **d** Charge density difference with isosurface of 0.008 e Å^−3^. **e** Projected density of states profiles of Cs and N ions for the most favorable adsorption configuration of Star-TrCN on the CsPbI_3_-PQD surface (V_I_-CN). **f** XPS Cs 3*d* core-level spectra of CsPbI_3_-PQD films with and without Star-TrCN post-treatment

We performed DFT-based optimized structure, molecular dynamic modeling and X-ray photoelectron spectroscopy (XPS) measurements to clarify this robust interaction between PQDs and Star-TrCN. According to the results of the DFT calculation (Fig. 2a, c), In the ESP analysis, Star-TrCN exhibited negative potential in the edge regions because of a strong electron lone pair existing at the terminal unit (–CO, –CN and –Cl). The negative potentials and distorted 3D star-shaped structure effectively can passivate the surface defects of the PQDs. In particular, substantial negative potential of Star-TrCN could form a chemical bond with the PQD surface. To verify this chemical bond, we constructed the PQD slabs and simulated the adsorption behavior of the Star-TrCN. The CsPbI_3_-PQD slab models are constructed using (001) surface that has two distinct terminations, CsI and PbI_2_ [45]. Then, we modeled the adsorption behavior of Star-TrCN on the PQD surfaces with the segmented molecule with functional groups (–CO, –CN and –Cl) (Fig. S7). The adsorption energy is summarized in Table S1 using the equation *E*_Ads_ = *E*_total _− *E*_slab _− *E*_Star-TrCN_. The most favorable configuration is –CN functional group of Star-TrCN on the Iodine vacancy of CsI termination with an adsorption energy of − 0.72 eV. Figure 2d shows the electron interaction between the two Cs atoms and –CN is the most significant. Since the iodine site is vacant, the –CN functional group directly interact with two Cs cations. The projected density of states of Cs and N atoms shows strong hybridization as shown in Fig. 2e, which indicates the covalent bond is formed between Cs ion and N atoms. This is consistent with the XPS core-level spectra in Star-PQD; the Cs 3*d*_3/2_ and Cs 3*d*_5/2_ signals were shifted to lower binding energies compared to those of the bare PQD film (by approximately 0.2 and 0.4 eV in the Cs 3*d* spectra, see Fig. 2f). This remarkable negative shift suggests the change of chemical bonding after Star-PQD films and the passivation effect of Star-TrCN, which is consistent with the DFT calculation. Furthermore, this Cs-N covalent bond can be plentiful considering the surface structure of PQDs. The CsI termination is more favoarble than PbI_2_ termination [46], and iodine vacancy can be largely generated with its low vacancy formation energy of 0.38 eV. This means the –CN functional group of the Star-TrCN can be easily linked with the PQDs by forming the Cs-N covalent bonds.

### Morphology Analysis of Star-TrCN Incorporated CsPbI_3_-PQDs

**Fig. 3:**
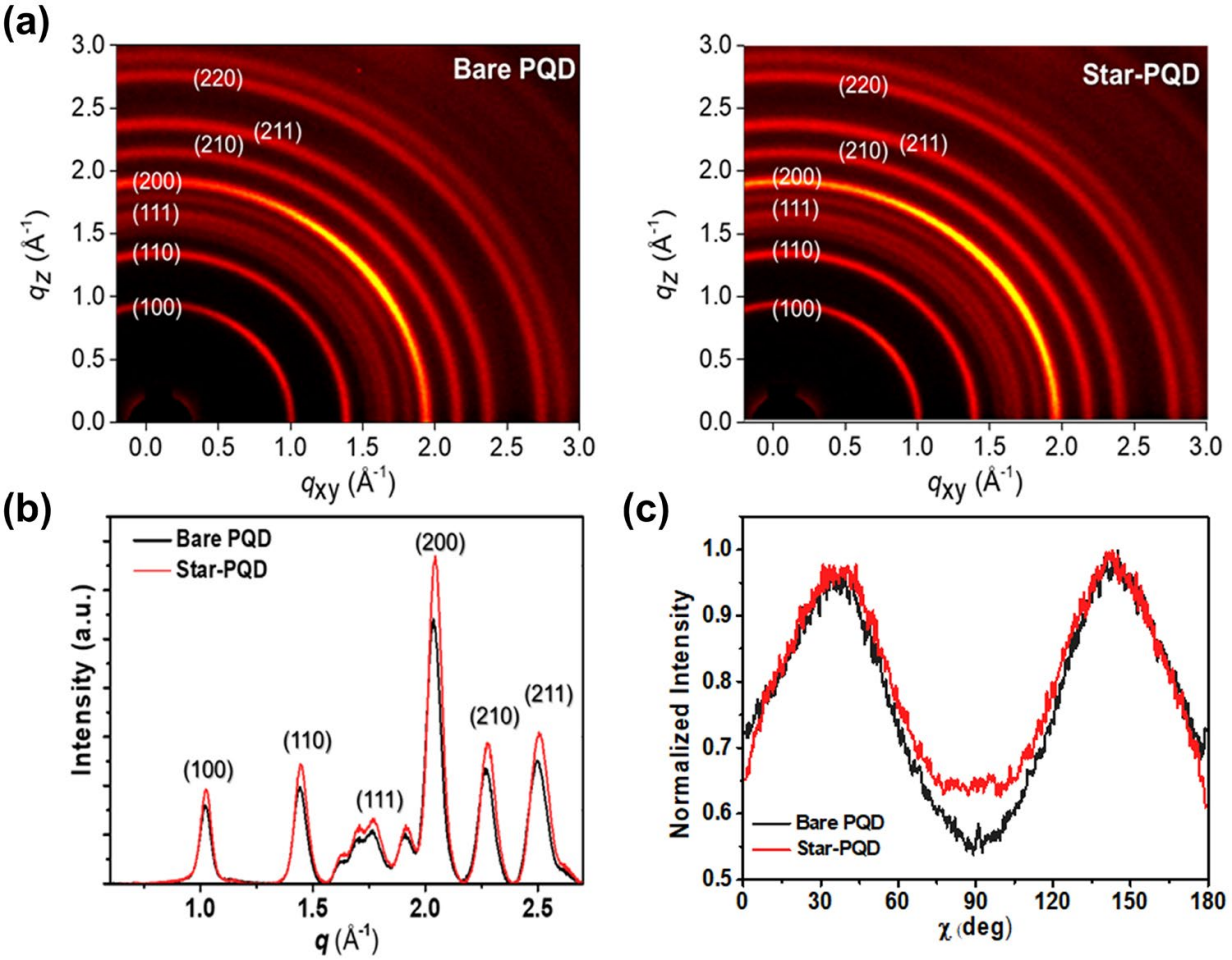
**a** 2D-GIWAXS patterns, **b** azimuthally integrated profiles and **c** azimuthal angle scans of the (200) peaks in the GIWAXS patterns of the control CsPbI_3_-PQD and Star-PQD hybrid films

Understanding the micromorphology of HPQD-type systems is crucial for comprehending the mechanism for enhancing device performance. Therefore, we determined the crystal structure of each morphology from synchronous 2D GIWAXS and AFM measurements. Figure 3a shows the GIWAXS diffraction patterns of the CsPbI_3_-PQD films in the bare and hybrid forms, respectively. Figure 3b shows the circular average profile of each film. The pristine CsPbI_3_-PQD film presented strong X-ray characteristic diffraction peaks characterizing the (100), (110), (111), (200), (210), (211) and (220) planes of cubic-phase CsPbI_3_. The pattern of the Star-PQD film was similar and the position of each diffraction peak was almost unchanged, suggesting that the cubic-phase of CsPbI_3_-PQD was maintained after HPQD formation. In addition, the 2D diffraction peaks distributed along azimuthal angles between 0° and 180° along the ring at *q* = 10 nm^−1^. Interestingly, the Star-PQD film featured a more ring-like diffraction pattern than the pristine CsPbI_3_-PQD film (Fig. 3a). This morphological change indicates that the Star-TrCN contributed to increased random orientation in the PQD film via a strong molecular interaction between the Star-TrCN and the PQDs. The increased random orientation can enhance the charge transport along the multiple directions (Fig. 3c) [47, 48]. We also measured the GIWAXS of Star-TrCN neat films to investigate the correlation between their dimension and PQDs. In contrast to linear-type organic semiconductors (e.g., ITIC, Y6 and PBDB-T) with a strong crystal orentation in other HPQD systems, the Star-TrCN showed a disordered random orientation (Fig. S8) [20, 33, 49]. This imply amorphous morphology of Star-TrCN can facilitate the incorporation of PQDs [50, 51].

From the AFM measurements, we obtained the surface morphology of the PQD film in contact with the HTM. In contrast to the bare PQD film, the Star-PQD film showed an additional intermediate layer that apparently covered the surface of the PQD layer (Fig. S9). The increased surface roughness of the Star-PQD thin film was attributed to the amorphous property of Star-TrCN. Additionally, the hydrophobic organic layer formed on the PQD surface effectively suppressed the moisture penetration. To evaluate the change in surface hydrophobicity, we measured the contact angles of bare Star-TrCN, bare PQD, Star-PQD and spiro-OMeTAD films coated on a glass substrate (Fig. S10). The contact angles of the bare Star-TrCN and PQD films were 102.1° and 59.8°, respectively. When Star-TrCN was treated on the PQD solids after the ligand exchange process, the contact angle of Star-PQD film was measured as 87.3°, higher than that of bare PQD solids.

### Cubic-Phase Stability of Star-TrCN Incorporated CsPbI_3_-PQDs

**Fig. 4:**
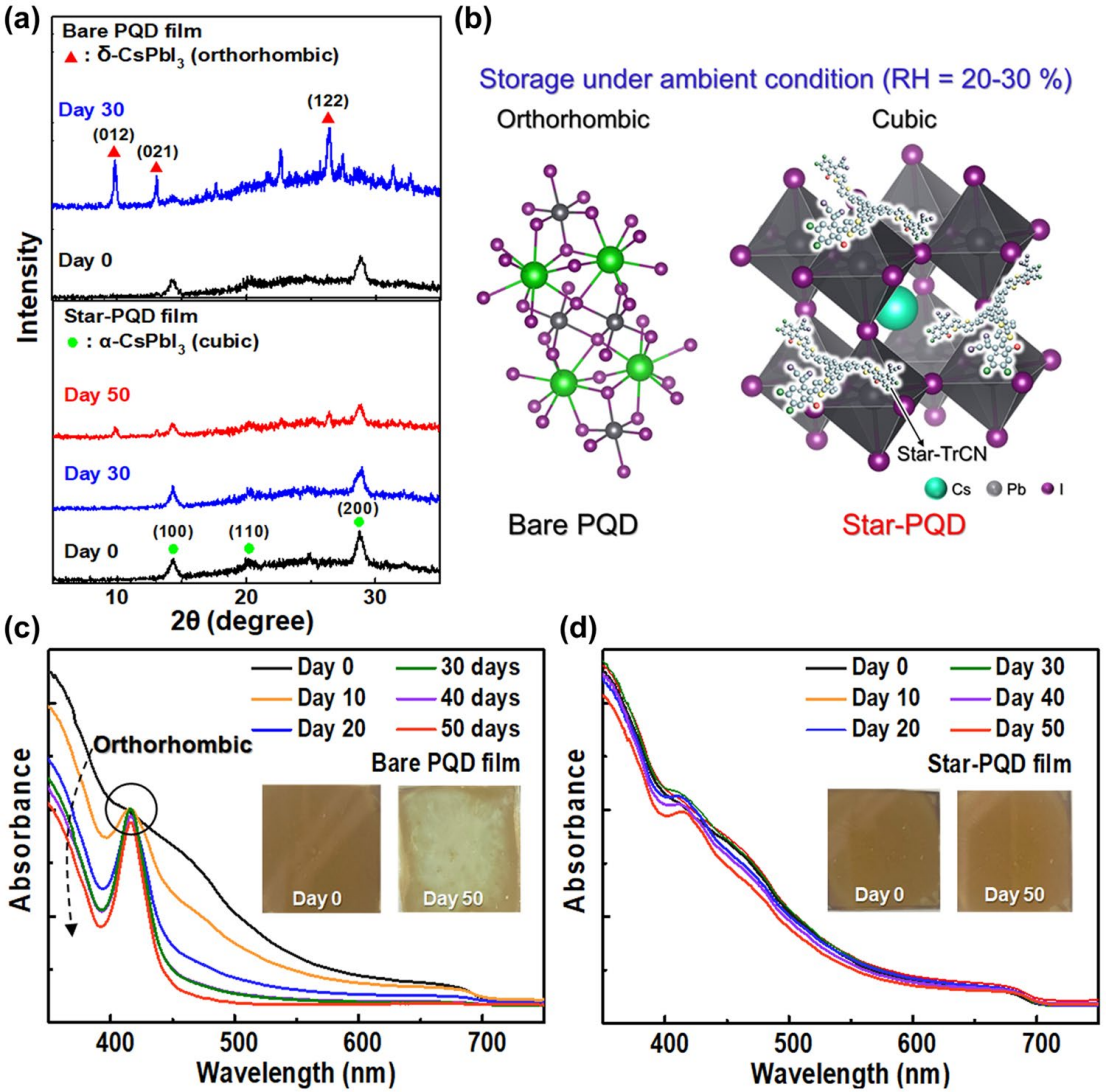
**a** XRD patterns of the glass/bare PQD film and the glass/Star-PQD film before and after aging for 50 days under ambient conditions (20–30% RH). **b** Schematic showing the cubic-phase degradation of CsPbI_3_-PQDs at 20–30% RH. UV–Vis absorption spectra of **c** glass/bare PQD film and **d** glass/Star-PQD film before and after aging for 50 days at 20–30% RH (Insets: film images before and after aging for 50 days at 20–30% RH)

Next, the elemental depth distribution in the integrated hybrid film was determined by time-of-flight secondary ion mass spectrometry (ToF-SIMS) (Fig. S11). The ToF-SIMS profiles of bare PQD and Star-PQD hybrid films detected the Pb and I ions constituting perovskite. Meanwhile, the simultaneous indication of C, N, S and Cl in the profile of the Star-PQD hybrid film confirmed the presence of Star-TrCN on the PQD surfaces. This indicated that the integrated Star-PQD layer was approximately 70–80 nm from the PQD surface, as shown in Fig. 1d, e. To confirm whether this integrated organic/PQD film practically contributed to the cubic-phase robustness of the CsPbI_3_-PQDs film, we conducted aging tests of the bare PQD and Star-PQD films under ambient conditions with a 20%–30% relative humidity (RH) at room temperature. The difference in cubic-phase stability between the CsPbI_3_-PQD films with and without the Star-TrCN treatment under ambient conditions was evaluated from X-ray diffraction (XRD) patterns of the two films (Fig. 4a). Initially, the spectra of both films exhibited the (100), (110) and (200) plane peaks of cubic-phase CsPbI_3_-PQDs. After 30 days, the intensities of the cubic-phase peaks were almost preserved in the Star-PQD film but were significantly reduced (being replaced by new orthorhombic-phase peaks) in the bare PQD film. After 50 days, the intensities of the cubic-phase peaks of CsPbI_3_-PQDs remained apparent in the Star-PQD film. This result indicated that Star-TrCN treatment significantly improves the cubic-phase stability of CsPbI_3_-PQDs by preventing moisture-induced hydration (Fig. 4b). Similarly, the UV–Vis absorbance of the bare PQD film was significantly reduced after aging for 20 days (Fig. 4c), whereas that of the Star-PQD film was highly preserved without any reduction even after aging for 50 days (Fig. 4d). As shown in photographic images (inset images in Fig. 4c, d), the Star-PQD film maintained its initial brownish color for 50 days under ambient conditions. However, the bare PQD film became yellowish and then transparent after 50 days (Fig. S12), implying that the cubic-phase of the CsPbI_3_-PQDs was completely transformed to the orthorhombic phase. In other words, the cubic-phase stability of CsPbI_3_-PQDs was much higher in the Star-PQD film than in the bare PQD film.

### Photophysical Properties and Photovoltaic Performance

**Fig. 5:**
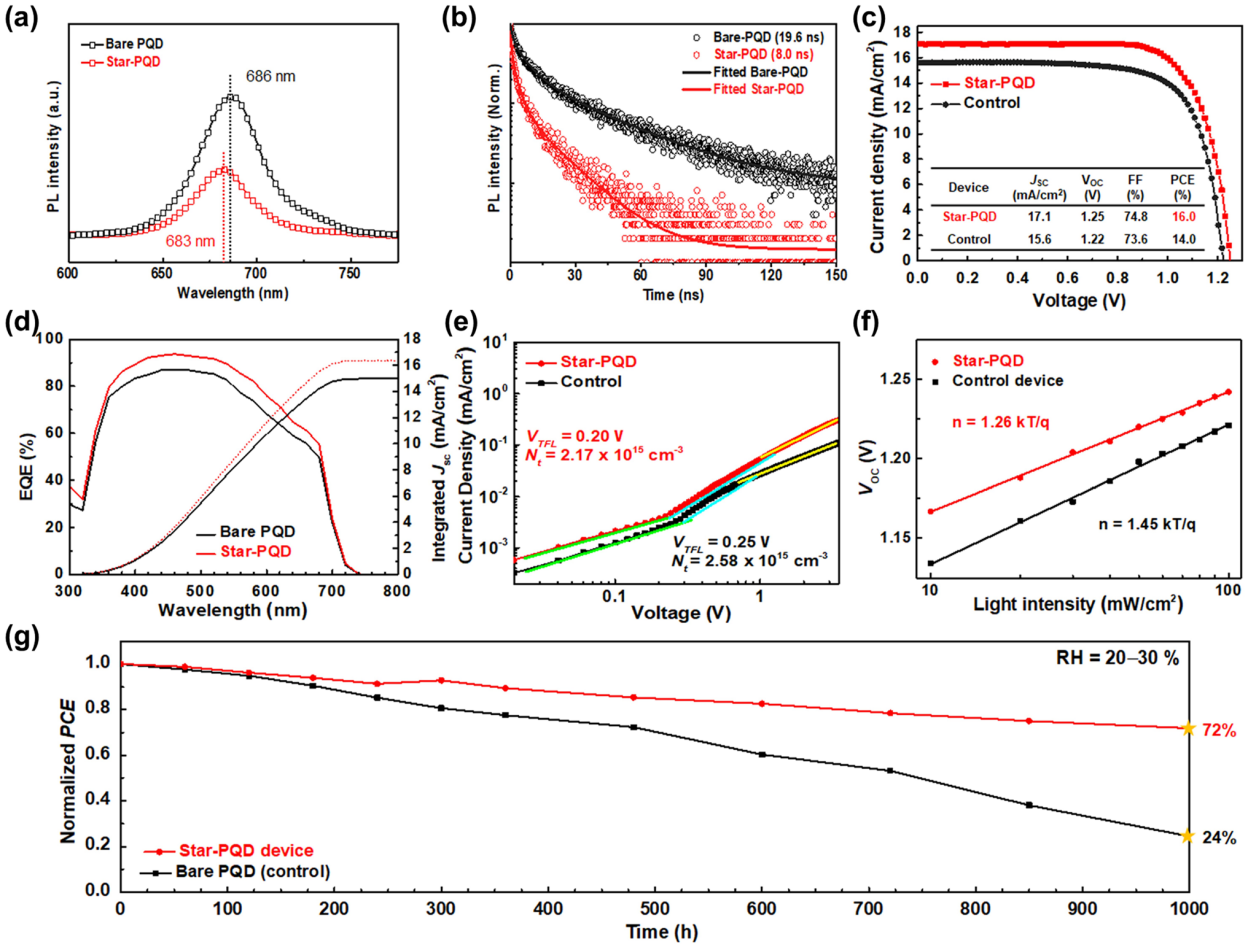
**a** PL spectra of glass/bare PQD and glass/Star-PQD films. **b** Time-resolved PL decay curves of the glass/bare PQD/HTM and glass/Star-PQD/HTM films. **c** Current density*‒*voltage curves, **d** EQE spectra and integrated *J*_SC_ of the best performing solar cells comprising bare PQD (control) and Star-PQD films. **e** SCLC fitting results obtained from dark *J*‒*V* measurements of hole-only devices compromising Star-PQD and bare PQD: trap density. **f** Light intensity-dependent *V*_OC_ of the Star-PQD- and bare PQD (control)-based solar cells. **g** Stability tests of solar cells fabricated from the bare PQD (control) and Star-PQD films under ambient conditions (20–30% RH)

To investigate the dynamic charge carrier transfer behavior after Star-TrCN treatment on PQDs, we carried out steady-state PL and TRPL measurements. As shown in Fig. 5a, the Star-PQD films showed more remarkable emission quenching than the bare PQD film, suggesting that they can achieve effective hole extraction. In addition, the Star-PQD films showed blueshift of the PL peak at 686–683 nm. This blueshift indicates the defect passivation effect of the Star-TrCN [41, 42]. The PL decay curves in Fig. 5b were fitted by a bi-exponential decay function and the detailed parameters are shown in Table S2. The carrier lifetime was much shorter in the Star-PQD/Spiro-OMeTAD film (8.0 ns) than in the bare PQD/Spiro-OMeTAD film (19.6 ns). These results indicate that the favorable band alignment at the Star-PQD/Spiro-OMeTAD interface reduced the carrier recombination loss and thus improved the efficiency of hole extraction.

Finally, we fabricated CsPbI_3_-PQD solar cells with and without Star-TrCN treatment of PQD solids (Fig. 1a). The fabrication method is described in Experimental section. The current density–voltage (*J*–*V*) curves of the Star-PQD and bare PQD-based devices are shown in Fig. 5c. The PCE of the Star-PQD-based device was improved to 16.0%, much higher than that of the bare PQD-based device (14.0%) with the same architecture and significantly higher than those of previously reported organic/CsPbI_3_-PQD HSCs (Table S3 and Fig. S13). The enhanced PCE of the Star-PQD-based devices was attributed to the improved short-circuit current density (*J*_SC_) and open-circuit voltage (*V*_OC_) caused by the cascade energy band structure at the Star-PQD/HTM interface and the defect passivation effect. The photovoltaic parameters of the best performing devices are detailed in Fig. 5c (inset table). Histograms of different parameters for the device are presented in Fig. S14, which suggests the excellent reproducibility of Star-PQD-based devices. The external quantum efficiency (EQE) and integrated *J*_SC_ spectra are shown in Fig. 5d. The fully integrated *J*_SC_ values of the Star-PQD- and bare PQD-based devices were 16.4 and 15.1 mA cm^−2^, respectively, consistent with the obtained values of the *J‒V* measurements.

The trap-state densities obtained from the dark *J‒V* measurements of hole-only devices comprising Star-PQD and bare PQD are shown in Fig. 5e. The Star-PQD-based device showed a lower trap-state density (*N*_t_ = 2.17 × 10^15^ cm^−3^) than the bare PQD device (*N*_t_ = 2.58 × 10^15^ cm^−3^), suggesting the high *V*_OC_ values of the Star-PQD-based device [52]. This result indicates that Star-TrCN could effectively passivate defects in CsPbI_3_ films, in consistent with XPS and steady-state PL results. In addition, we compare hole mobility of Star-PQD and bare PQD (Fig. S15). The hole mobility (*μ*_h_) was estimated by using the SCLC model [53]. The hole mobility of Star-PQD increased from 3.03 × 10^−5^ to 9.89 × 10^−5^ cm^2^ V^−1^ s^−1^, indicating that the Star-TrCN could effectively reduce the defect density in CsPbI_3_ films, in consonance with XPS and steady-state PL results. The light-dependent *V*_OC_ measurement was performed to compare the charge carrier recombination in solar cells based on Star-PQD and bare PQD (Fig. 5f). The slope of the Star-PQD device (1.26 *kT*/*q*) was lower than that of the bare PQD device (1.45 *kT*/*q*). This result indicated that trap-assisted recombination was effectively decreased in Star-PQD devices [54]. Figure S16 shows the hysteresis behaviors of the Star-PQD- and bare PQD-based devices. The pronounced hysteresis effect in the bare PQD device was suppressed in the Star-PQD-based device. In the forward scan, the initial PCE of Star-PQD-based device was maintained up to 90%, whereas that of bare PQD-based device was maintained only up to 79%. This result indicates that charge accumulation at the PQD/HTM interface was reduced in the Star-PQD-based devices (by virtue of the efficient hole extraction ability as mentioned above). The operational device stability of the Star-PQD solar cells was demonstrated in stable power output measurements (Fig. S17). The long-term stabilities of solar cells comprising bare PQD and Star-PQD were compared after shelf storage at the same ambient conditions (25 °C, 20–30% RH) over 1000 h without additional encapsulation (Fig. 5g). The Star-PQD device was remarkably more stable than bare PQDs, consistent with our experimental results on moisture stability with and without the Star-TrCN treatment (Fig. 4). This result indicated that the Star-PQD hybrid approach improved the moisture stability of cubic-phase CsPbI_3_-PQDs, which have an appropriate bandgap energy for single-junction solar cells. Consequently, the best performing devices in this study showed a PCE of 16.0% and retained > 72% of the initial efficiency after 1000 h.

## Conclusions

In summary, we fabricated efficient and stable CsPbI_3_-PQDs by combining organic conjugated molecules with PQDs. To achieve a successful hybrid system, we newly designed a 3D star-shaped semiconductor material (Star-TrCN) with an excellent geometric balance of coplanarity and distortion characteristics. The twisted 3D structure of Star-TrCN inhibits self-aggregation, increasing the compatibility between the organic semiconductors and PQDs. The Star-PQD hybrid film significantly enhances the cubic-phase stability of CsPbI_3_-PQDs by passivating the surface defect and preventing moisture penetration through hygroscopic HTM (Spiro-OMeTAD). Consequently, the initial PCE of the HPQD device was maintained at 72% even after 1000 h. Furthermore, Star-TrCN forms a cascaded energy band structure, boosting the charge extraction efficiency and achieving 16.0% PCE. We report multifunctional 3D star-shaped organic semiconductors to enable efficient and stable PQD solar cells. This multidimensional organic semiconductor design can potentially realize commercial PQD photovoltaics.

## Supplementary Information

Below is the link to the electronic supplementary material.

Supplementary file1 (PDF 2037 KB)
