# Supplementary material for: High-Performance Perovskite Quantum Dot Solar Cells Enabled by Incorporation with Dimensionally Engineered Organic Semiconductor
Source: Nanomicro Lett. 2022 Oct 17;14:204. doi: 10.1007/s40820-022-00946-x (PMC9576836; doi:10.1007/s40820-022-00946-x)
Supplement: Supplementary file 1 — Supplementary file1 (PDF 2037 KB) [file 40820_2022_946_MOESM1_ESM.pdf]

Supporting Information for

High-Performance Perovskite Quantum Dot Solar Cells Enabled by Incorporation with Dimensionally Engineered Organic Semiconductor

Seyeong Lim[1, ‡], Dae Hwan Lee[1, ‡], Hyuntae Choi[1], Yelim Choi[1], Dong Geon Lee[2, 3], Sung Beom Cho[3, 4], Seonkyung Ko[5], Jongmin Choi[5, *], Younghoon Kim[6, *], Taiho Park[1, *]

[1]Department of Chemical Engineering, Pohang University of Science and Technology (POSTECH), Pohang 37673, Republic of Korea

[2]Department of Materials Science and Engineering, Hanyang University Seoul 04763, Republic of Korea

[3]Center of Materials Digitalization, Korea Institute of Ceramic Engineering and Technology (KICET) Jinju 52851, Republic of Korea

[4]Department of Materials Science and Engineering, Ajou University, Suwon, 16499 Republic of Korea

[5]Department of Energy Science and Engineering, Daegu Gyeongbuk Institute of Science and Technology (DGIST), Daegu 42988, Republic of Korea

[6]Department of Chemistry, Kookmin University, Seoul 02707, Republic of Korea

‡ Seyeong Lim and Dae Hwan Lee contributed equally to this work.

*Corresponding authors. E-mail: taihopark@postech.ac.kr (Taiho Park), whdals1062@dgist.ac.kr (Jongmin Choi), younghoon.kim@kookmin.ac.kr (Younghoon Kim)

S1 Synthetic Procedures and Characterization

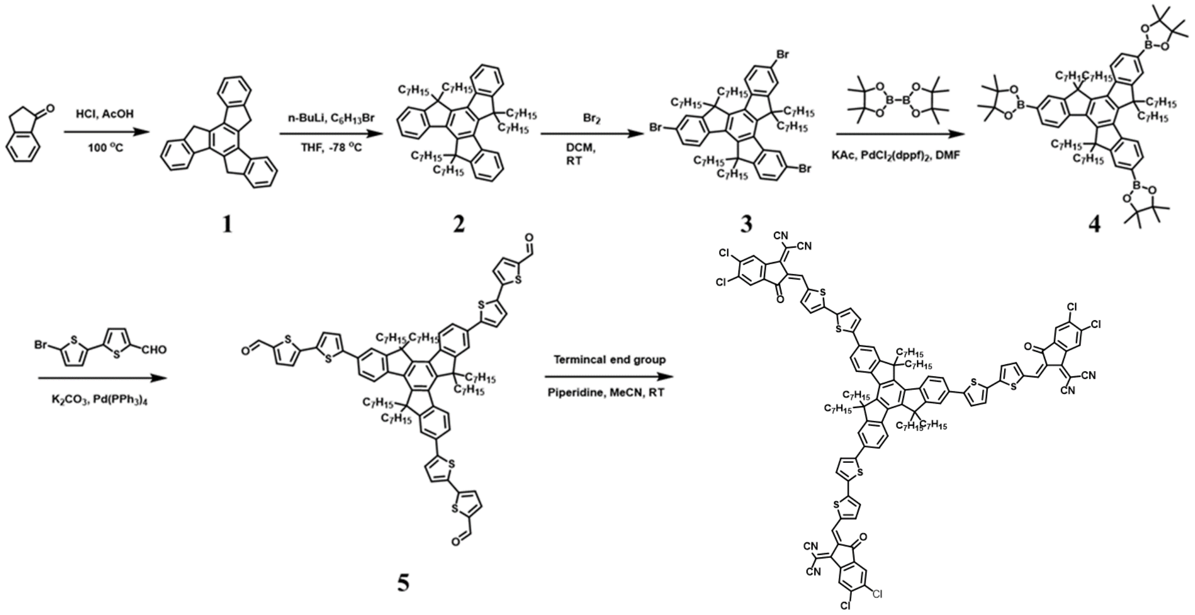

Scheme S1 Synthesis scheme for Star-TrCN

Synthesis of compound 1: 1-indanone (5 g, 23.8 mmol) was added to a mixed solution of acetic acid (60 mL) and concentrated hydrochloric acid (30 mL). Then the solution was heated to 100 °C for 24 h. After cooling to room temperature, the mixture was poured into 800 ml of ice water, and stirred for 1 h. The yellow precipitate was filtered, and washed with water, ethanol, cold acetone and cold dichloromethane. Compound 1 was obtained as light yellow solid (9.2 g,

63.9%). ^{1}H NMR (500 MHz, CDCl3, δ): 7.96 (d, J = 7.7 Hz, 3H), 7.70 (d, J = 7.4 Hz, 3H), 7.50 (d, J = 7.5 Hz, 3H), 7.41 (d, J = 7.3 Hz, 3H), 4.28 (d, *J* = 4.6 Hz, 6H).

Synthesis of compound 2: Compound 1 (5.0 g, 14.6 mmol) was dissolved in 120 mL dry THF under N_2 atmosphere. After cooling to -78 °C, n-BuLi (73 mL, 146.02 mmol, 2.4 M in n-hexane) was added dropwise and the mixture was stirred at -78 °C for 5 h. A solution of $C_7H_{15}Br$ (31.54 g, 17518.22 mmol) in 50 mL THF was injected slowly. The mixture was slowly warmed to room temperature and stirred overnight. Then the solution was quenched with saturated ammonium chloride solution (150 mL), extracted with ethyl acetate, dried over $MgSO_4$. After the organic phase was concentrated under reduced pressure, the crude product was purified by column chromatography on silica gel using petroleum ether as the eluent. Compound 2 was obtained as off-green solid (9.78 g, 72.1%). ^{1}H NMR (600 MHz, Chloroform-*d*) δ 8.36 (d, 3H), 7.46 (d, *J* = 7.2 Hz, 3H), 7.38 (p, *J* = 7.2 Hz, 6H3.04 – 2.89 (m, 6H), 2.16 – 2.00 (m, 6H), 0.98 – 0.77 (m, 40H), 0.59 (t, J = 7.1 Hz, 18H), 0.48 (m, 12H). ^{13}C NMR (151 MHz, Chloroform-*d*) δ 153.65, 144.85, 144.83, 140.35, 138.38, 126.32, 125.94, 124.65, 124.63, 122.17, 55.62, 36.97, 31.49, 29.67, 29.49, 29.37, 23.89, 22.27, 22.21, 14.11, 13.86, 13.84.

Synthesis of compound 3: A compound 2 (8.72 g, 9.37 mmol) was dissolved into DCM (40 mL) was carefully degassed before bromine was added. A bromine (5.17 g, 32.79 mmol) was added dropwise under ice bath. The mixture was gradually warmed to room temperature and stirred for 48 h. After quenching with saturated $NaHSO_3$ aqueous solution, the mixture was extracted with chloroform for three times, and the organic phase was dried over $MgSO_4$. After the solvent was removed, the crude product was purified by column chromatography on silica gel (silica gel; eluent: hexane:DCM= 7:3) Compound 3 was obtained as pale yellow solid (8.1g, 74.3%) ^{1}H NMR (600 MHz, Chloroform-d) δ 8.17 (d, J = 8.4 Hz, 3H), 7.56 (d, J = 2.0 Hz, 3H), 7.51 (dd, J = 8.4, 2.0 Hz, 3H), 2.83 (m, 6H), 2.01 (m, 6H), 1.26-0.98(m, 40H), 0.55 (t, J= 7.2 Hz, 18H), 0.51 – 0.41 (m, 12H), ^{13}C NMR (151 MHz, Chloroform-d) δ 155.90, 144.94, 138.88, 137.64, 129.39, 125.92, 125.55, 121.07, 55.98, 36.75, 31.47, 29.71, 29.62, 28.84, 23.89, 22.48, 14.12, 13.88, 13.85.

Synthesis of compound 4: A mixture of compound 3 (3.0 g, 3.22 mmol), bis(pinacolato)diboron (4.91 g, 19.34 mmol), AcOK (2.46 g, 24.51 mmol), and DMF (90 mL) was carefully degassed before $Pd(dppf)_2Cl_2$ (150 mg, 0.246 mmol) was added. The mixture was stirred for 48 h at 90 °C under N_2. After the solution was cooled to room temperature, water (150 mL) and CH_2Cl_2 (150 mL) were added. The organic layer was separated and washed with water for three times. After removal of the organic solvent, the crude product was purified by column chromatography (silica gel; eluent: petroleum ether:ethyl acetate = 2:1) to afford compound 4 (2.57 g, yield: 60.9%) as a light yellow solid. ^{1}H NMR (400 MHz, Chloroform-*d*) δ 8.54 (d, *J* = 2.1 Hz, 3H), 8.16 (d, *J* = 2.1 Hz, 3H), 7.62 (d, J = 5.2 Hz, 3H) 2.77 – 2.75 (m, 6H), 2.02 (m, 6H), 1.26-0.89 (m, 52H), 0.55 (t, J= 7.2 Hz, 18H), 0.51 – 0.41 (m, 12H), ^{13}C NMR (151 MHz, Chloroform-*d*) δ 155.86, 145.98, 145.67, 145.29, 140.67, 138.36, 136.74, 129.61, 129.52, 129.48, 129.26, 129.21, 129.16, 127.29, 127.26, 125.96, 125.89, 125.84, 125.66, 125.62, 125.60, 123.10, 56.06, 55.99, 55.97, 55.89, 36.77, 36.72, 36.69, 36.66, 36.62, 31.47, 29.59, 29.58, 29.55, 29.53, 29.51, 28.82, 28.81, 28.79, 26.33, 23.93, 23.92, 23.87, 22.65, 22.50, 22.47, 13.88, 13.85, 13.68.

Synthesis of compound 5: 5'-bromo-[2,2'-bithiophene]-5-carbaldehyde (0.77 g, 2.82 mmol), compound 4 (0.62 g, 0.47 mmol), $Pd(PPh_3)_4$ (0.11 g, 0.096 mmol) and K_2CO_3 (4.98 g, 36.03 mmol) were dissolved in Toluene (20 mL) and the reaction mixture was heated at 120 °C overnight under Ar. After the reaction mixture was cooled to room temperature, the solution was quenched with water and extracted with CH_2Cl_2. The combined organic layer was washed with brine for several times, and then dried over anhydrous $MgSO_4$. After the solvent was removed off under reduced pressure, the crude product was purified by column chromatography (silica gel; eluent: petroleum ether: diethyl ether = 8:2) to afford compound 5 (0.51 g, yield: 72.1%) as an orange solid. ^{1}H NMR (600 MHz, Chloroform-*d*) δ 9.81 (d, *J* = 16.9 Hz, 3H),

8.33 (d, *J* = 8.2 Hz, 3H), 7.67 (m, 6H), 7.35 (q, *J* = 3.7 Hz, 3H), 7.33 (m, 3H), 7.31 (m, *J* = 3.9 Hz, 3H), 7.26 (m, *J* = 3.2 Hz, 3H), 2.90 (d, *J* = 5.1 Hz, 6H), 2.08 (d, *J* = 5.0 Hz, 6H), 0.99 – 0.72 (m, 40H), 0.58 -0.51 (m, 18H), 0.55 – 0.45 (m, `12H). ^{13}C NMR (151 MHz, $CDCl_3$) δ 182.47, 182.43, 154.59, 147.30, 146.66, 145.82, 145.65, 142.07, 141.58, 140.43, 137.96, 137.41, 137.26, 137.13, 135.17, 135.13, 135.10, 135.06, 134.91, 132.14, 132.08, 131.94, 131.75, 131.20, 130.51, 130.45, 130.37, 128.54, 128.46, 128.37, 128.06, 128.01, 127.97, 127.93, 127.87, 127.25, 127.09, 126.20, 125.10, 124.39, 124.26, 124.20, 124.13, 123.97, 119.28, 114.17, 77.22, 77.01, 76.80, 55.89, 37.00, 31.47, 31.41, 29.70, 28.85, 24.87, 23.98, 22.76, 22.48, 13.87, 13.84, 13.74. HRMS (MALDI$^+$) m/z: Calcd for $[C_{96}H_{114}O_3S_6 + H]^+$, m/z = 1509.2642. Found: m/z = 1508.2444 $[M+H]^+$

Synthesis of Star-TrCN: Compound 5 (0.25 g, 0.19 mmol) and 2-(5,6-dichloro-3-oxo-2,3-dihydro-1H-inden-1-ylidene) malononitrile (0.53 g, 1.9 mmol) were first dissolved in $CHCl_3$ (60 mL). Piperidine (four drops) was then added and the reaction mixture was stirred at 85°C overnight. After the reaction mixture was quenched with water and extracted with CH_2Cl_2, it was dried over anhydrous MgSO4. After the solvent was removed under reduced pressure, the crude product was purified by precipitation in methanol solvent and purified through filtration (0.21 g, yield: 52%) as a black solid. ^{1}H NMR (600 MHz, chloroform-d) ^{1}H NMR (600 MHz, chloroform-*d*) δ 8.28 (d, *J* = 8.1 Hz, 3H), 7.86 (m, 3H), 7.58 – 7.53 (m, 12H), 7.19 – 7.15 (m, 3H), 7.06 – 7.03 (m, 3H), 3.01 (s, 6H), 2.19 (s, 6H), 1.76-1.33 (m, 40H), 1.05 -0.89 (m, 18H), 0.68-0.60 (m, 12H), ^{13}C NMR (151 MHz, chloroform-*d*) δ 208.23, 134.35, 130.87, 125.36, 124.94, 51.35, 49.59, 37.09, 32.77, 31.84, 31.45, 29.96, 29.60, 29.24, 28.78, 27.02, 26.06, 25.86, 22.56, 22.36, 19.64, 13.87, 13.68. HRMS: Calcd for $[C_{148}H_{120}Cl_6N_6O_3S_6]^+$, m/z = 2243.51 Found: m/z = 2240.6067 $[M+H]^+$ Elemental Anal. calcd. for $C_{148}H_{120}Cl_6N_6O_3S_6$: C, 70.67; H, 7.39; Cl, 9.48; N, 3.75; O, 2.14; S, 6.58 Found: C, 71.19; H, 7.47; N, 3.9; S, 6.16

S2 Supplementary Figures and Tables

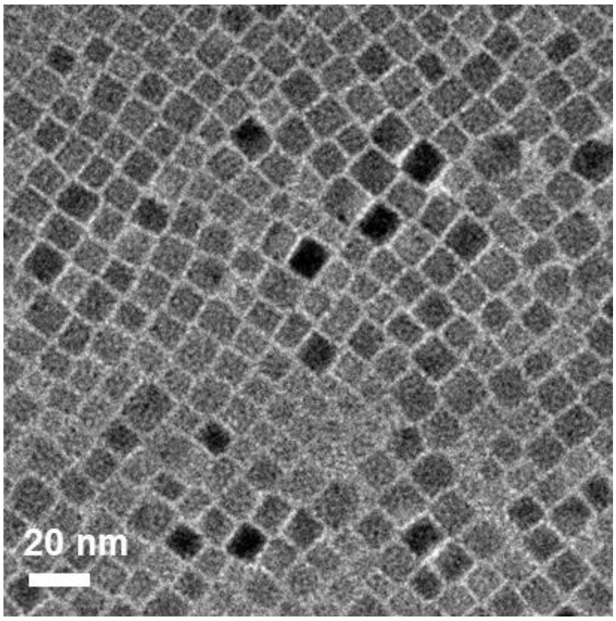

Fig. S1 HR-TEM image of $CsPbI_3$ PQDsSynthesis details of Star-TrCN

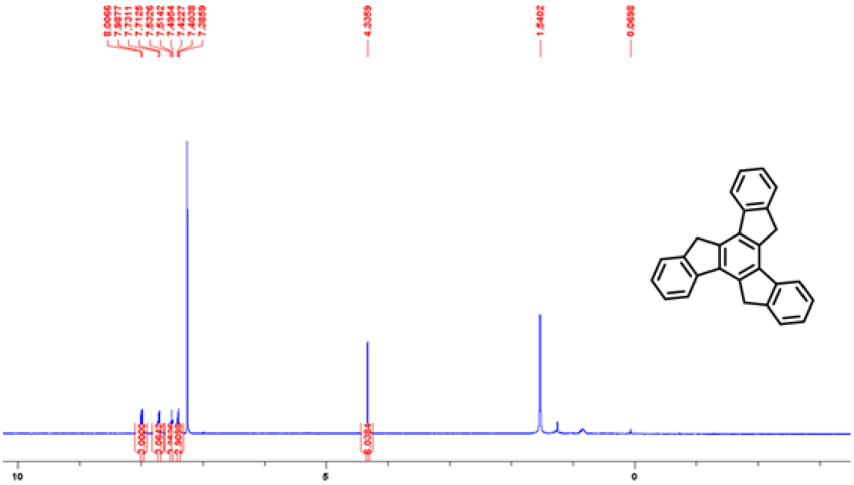

^{1}H NMR of compound 1

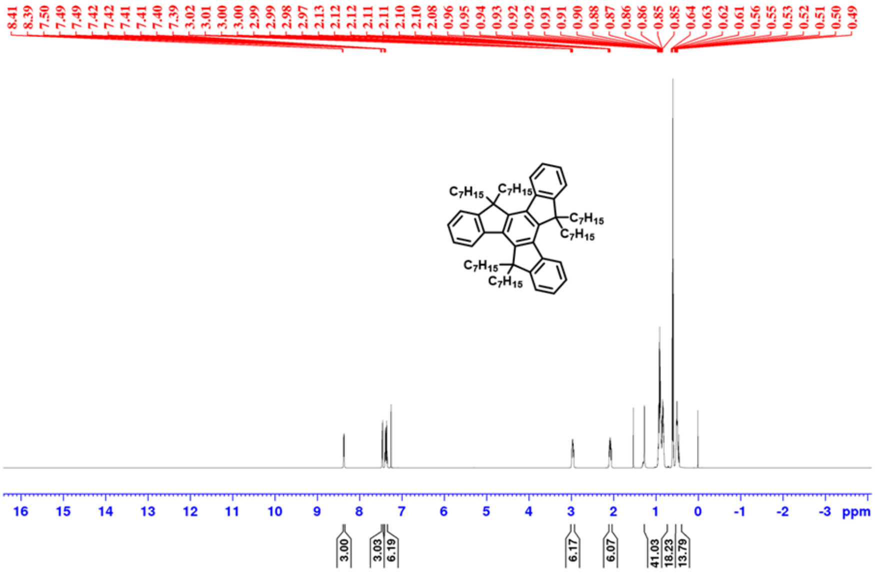

^{1}H NMR of compound 2

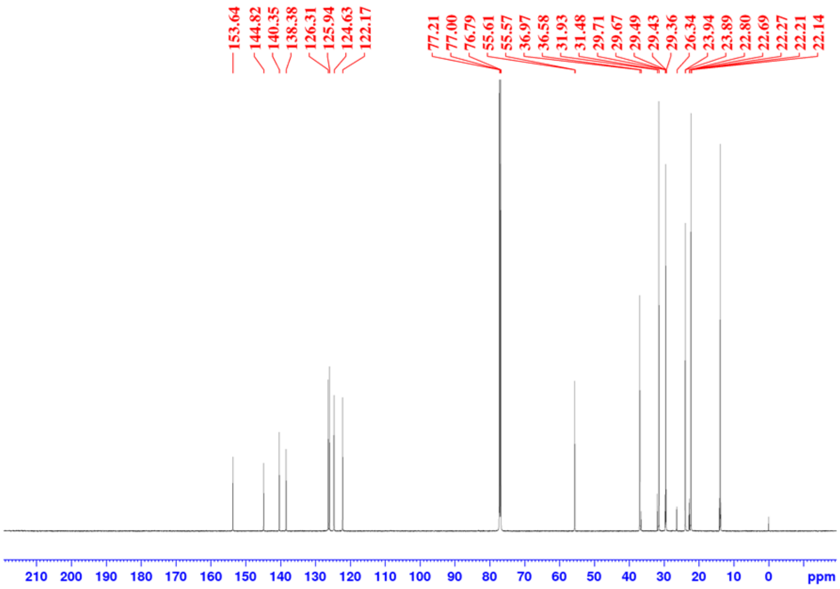

^{13}C NMR of compound 2

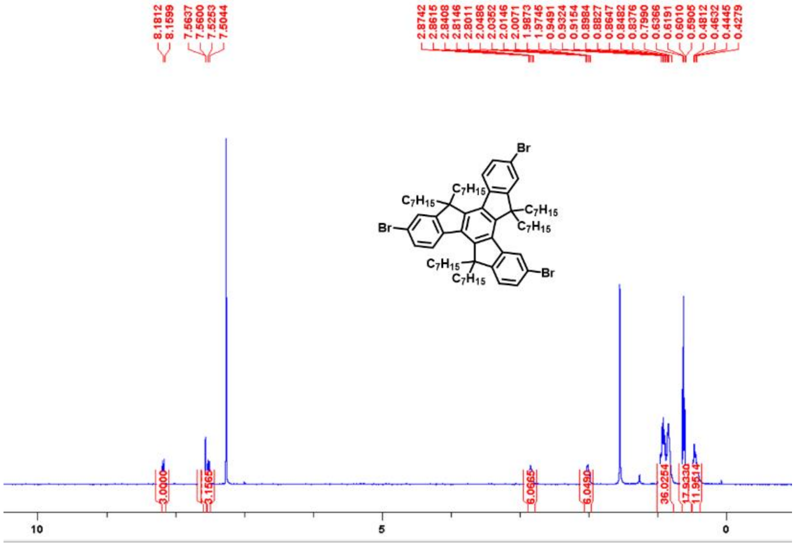

^{1}H NMR of compound 3

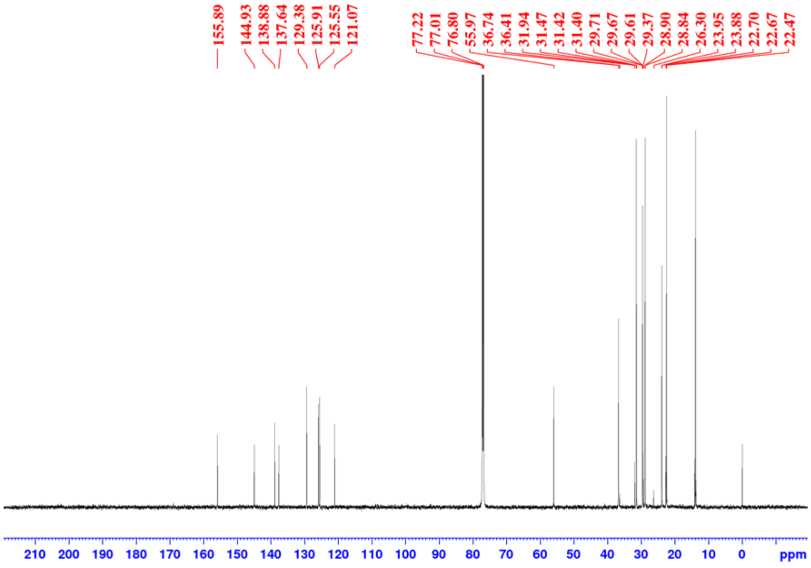

^{13}C NMR of compound 3

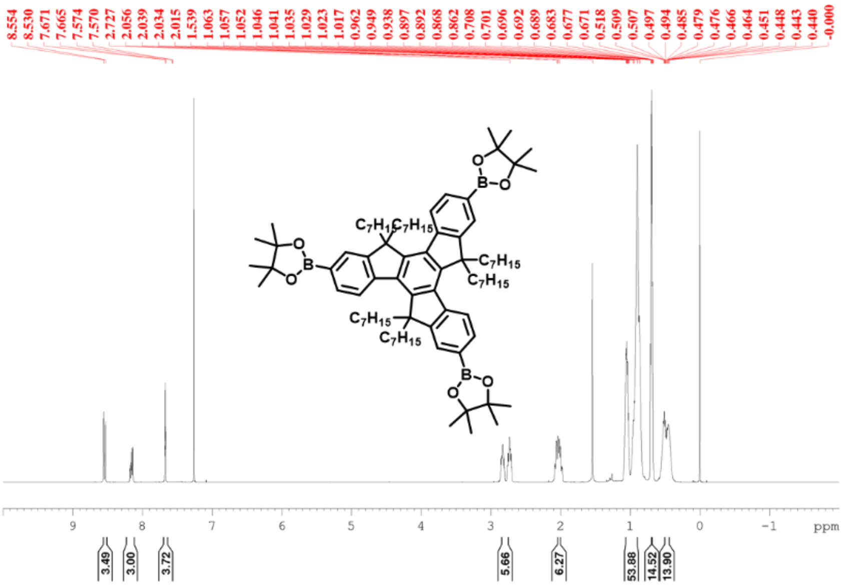

^{1}H NMR of compound 4

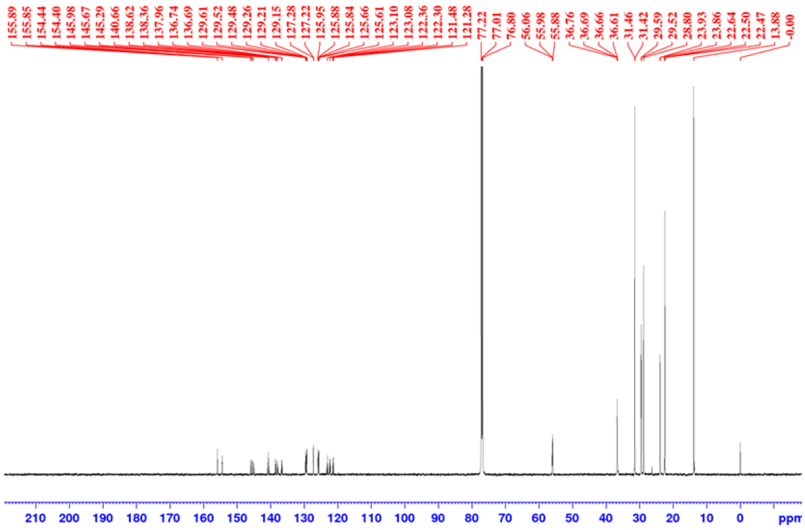

^{13}C NMR of compound 4

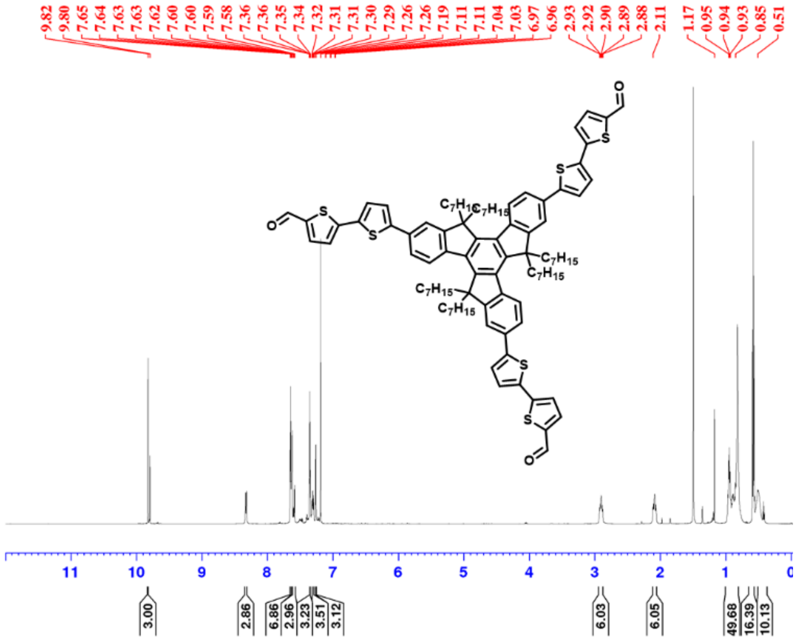

^{1}H NMR of compound 5

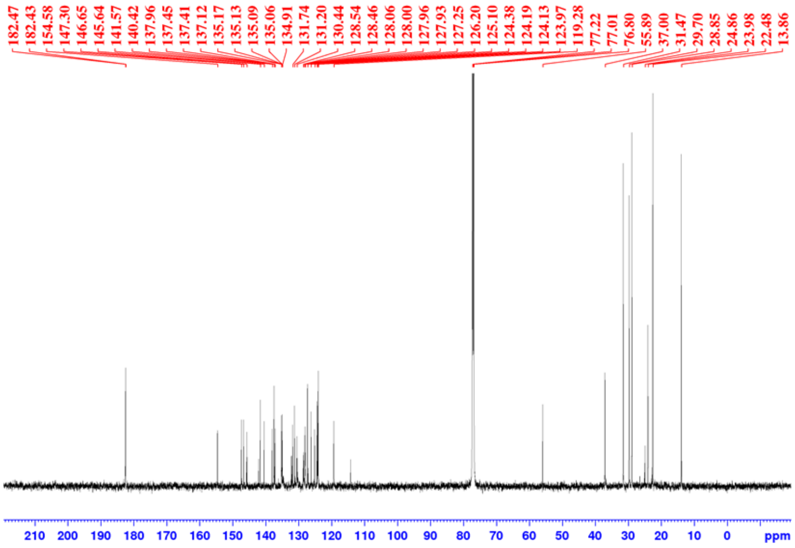

^{13}C NMR of compound 5

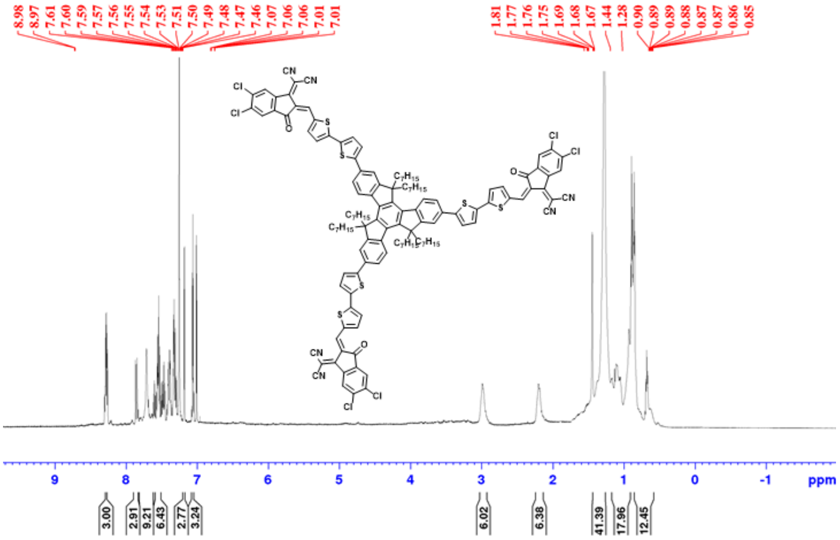

^{1}H NMR of Star-TrCN

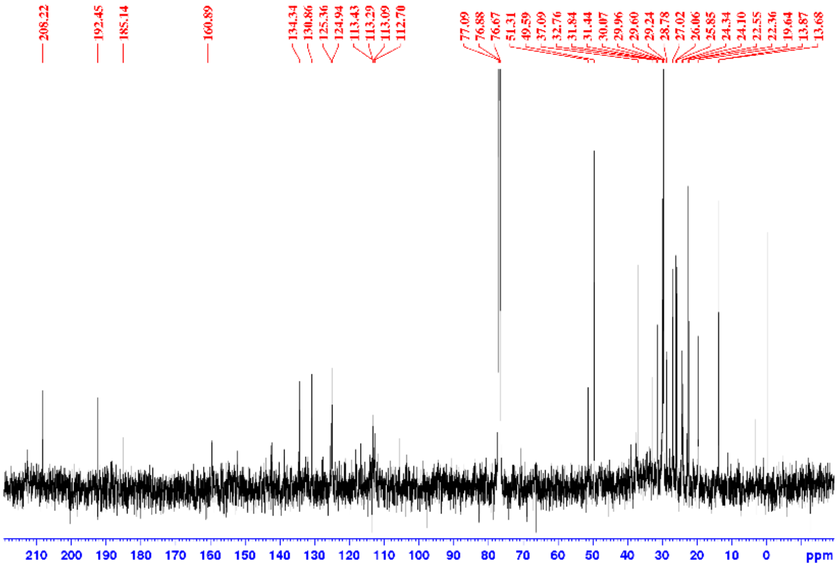

^{13}C NMR of Star-TrCN

Fig. S2 H^1 /C^{13} nuclear magnetic resonance (NMR) spectroscopy of all the compounds

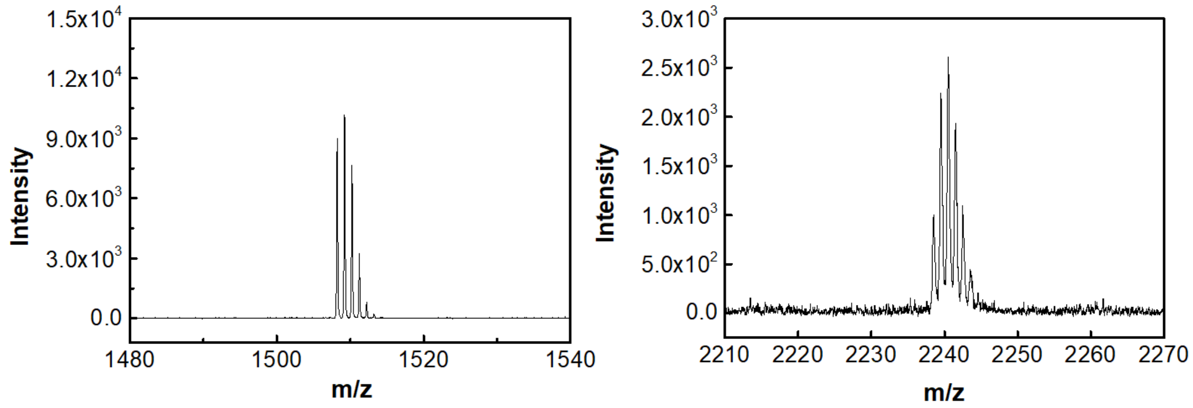

Fig. S3 Matrix associated laser desorption ionization - time of flight mass spectrometry (MALDI-TOF) of the compound 5 and Star-TrCN

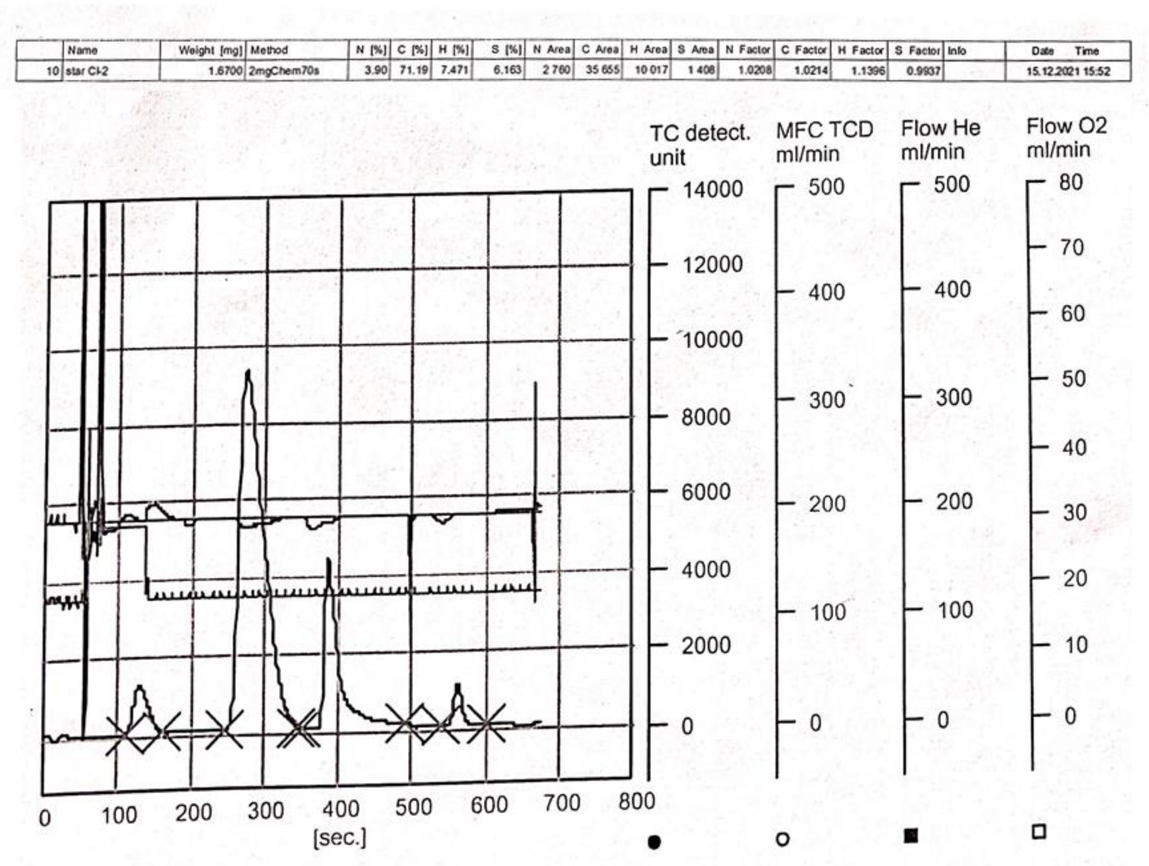

	Name	Weight [mg]	Method	N [%]	C [%]	H [%]	S [%]	N Area	C Area	H Area	S Area	N Factor	C Factor	H Factor	S Factor	Info	Date Time
10	star Cl-2	1.6700	2mgChem70s	3.90	71.19	7.471	6.163	2 760	35 655	10 017	1 408	1.0208	1.0214	1.1396	0.9937		15.12.2021 15:52

Fig. S4 Elemental analysis (EA) of the compounds of the Star-TrCN

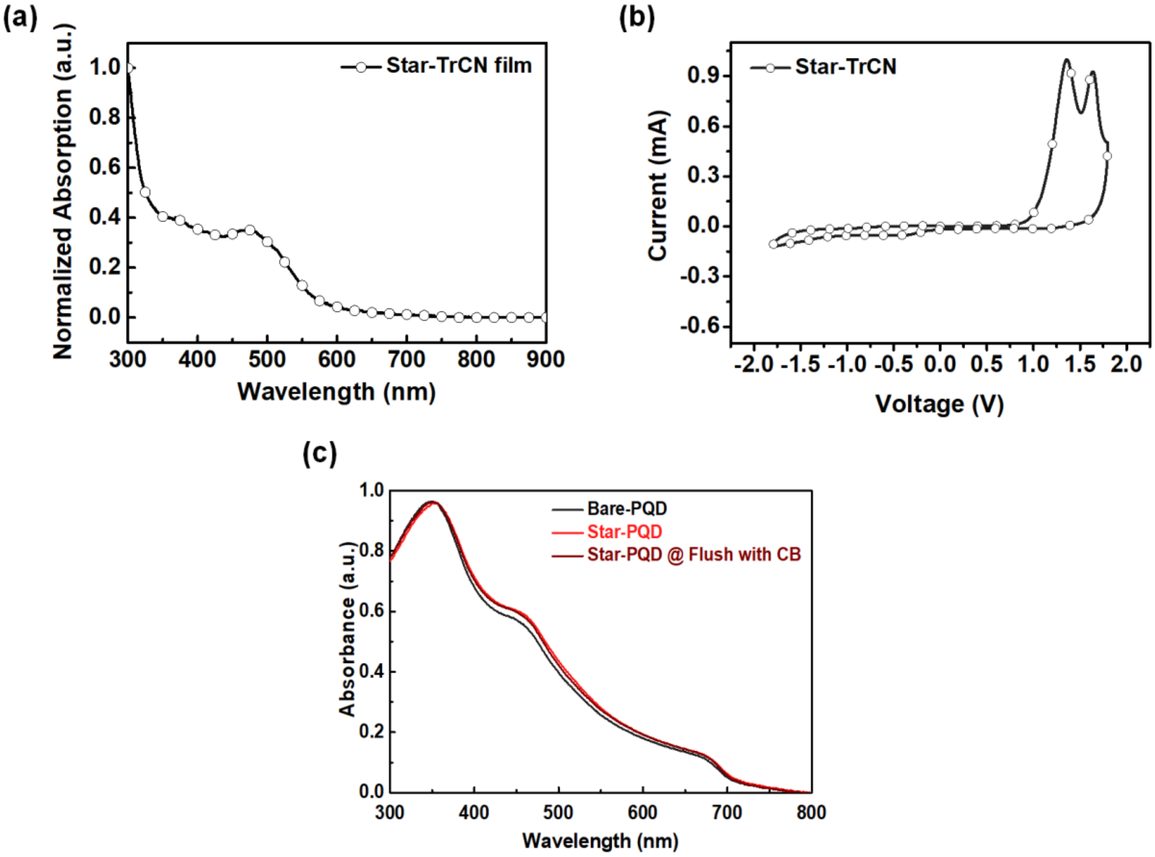

Fig. S5 (**a**) Ultraviolet–visible absorption spectrum, (**b**) Cyclic voltammogram (CV) of Star-TrCN and (**c**) UV-vis absorption spectra of bare PQD, Star-PQD, and Star-PQD after flushing with chlorobenzene

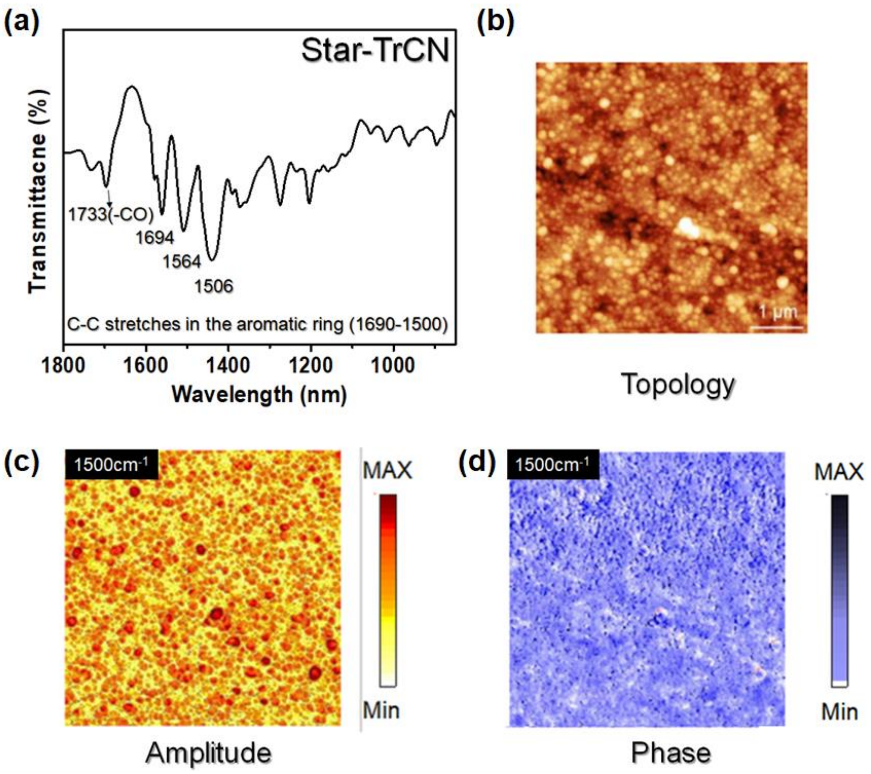

Fig. S6 Nano-FTIR spectroscopy experiments of Star-PQD layer after chlorobenzene flushing (**a**) AFM Topographic image. (**b**) FTIR spectral peak. (**c**) nano-IR absorption amplitude image at 1600 $cm^{-1}$and (**d**) Phase image, corresponding to the aromatic ring

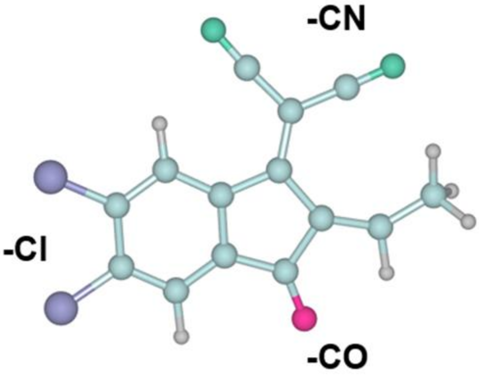

Fig. S7 Terminal unit of segment Star-TrCN

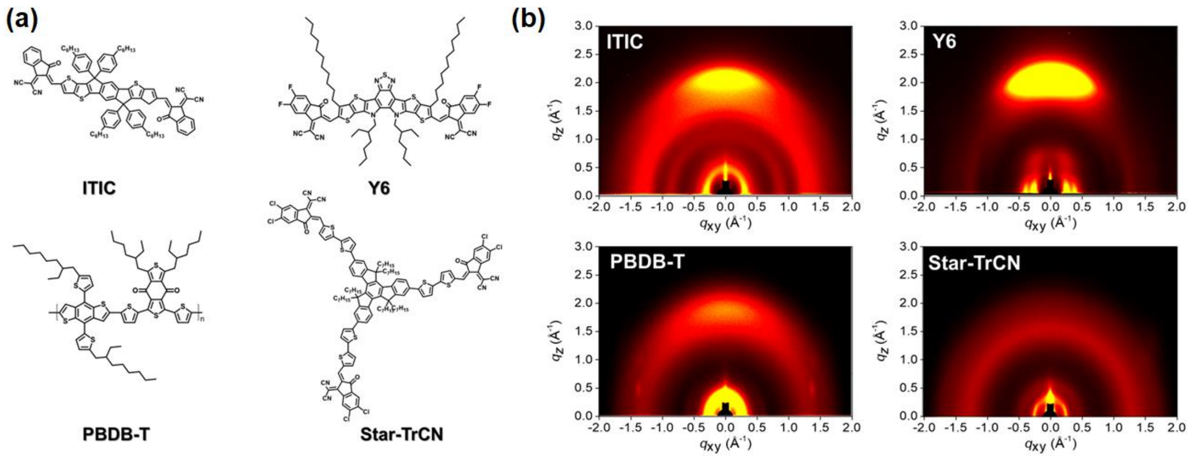

Fig. S8 (**a**) Chemical structure of linear 1D types of organic semiconductor(ITIC, Y6, PBDB-T) and 3D typs of Star-TrCN and (**b**) 2D-GIWAXS patterns of each neat films

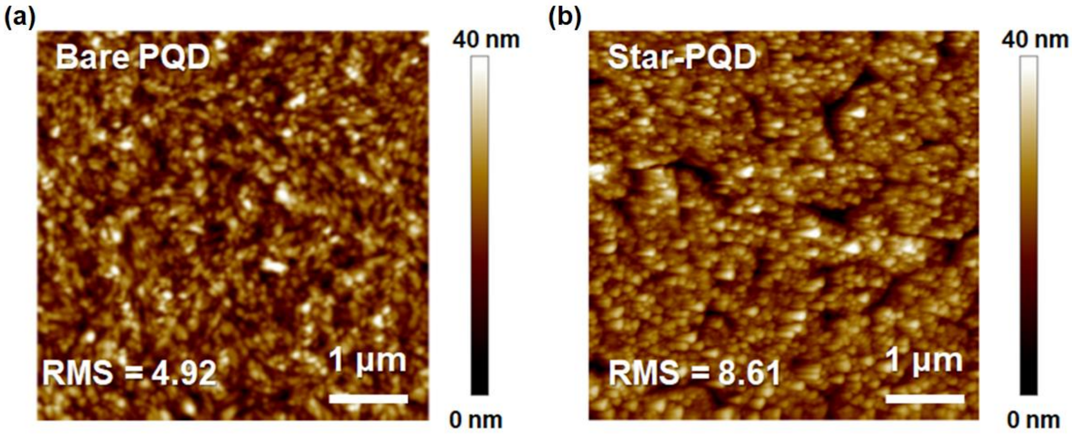

Fig. S9 AFM height images (5 × 5 μm^2) of bare PQD and Star-PQD films

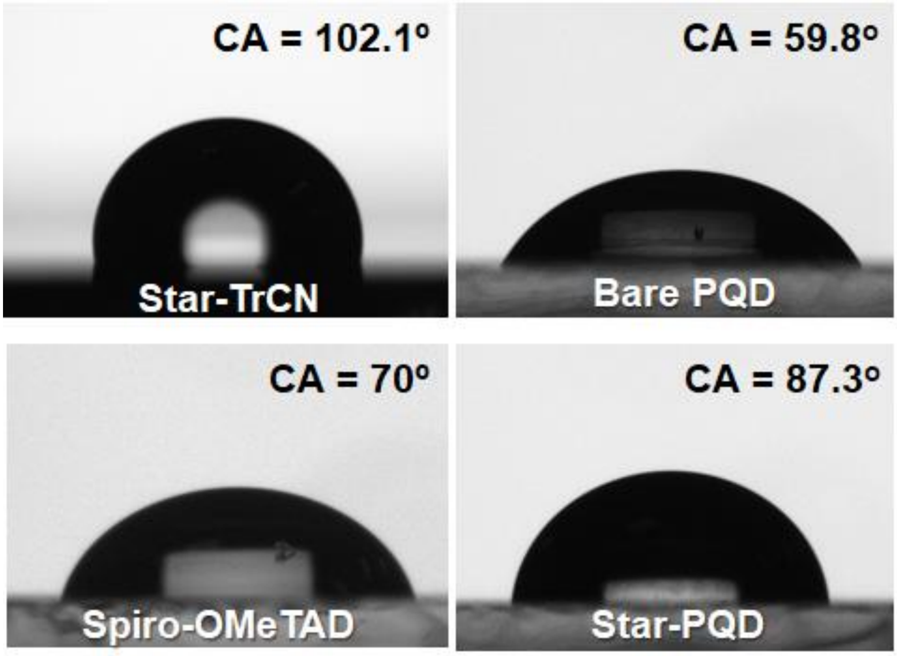

Fig. S10 Contact angles of water on bare Star-TrCN, bare PQD, spiro-OMeTAD, and Star-PQD films

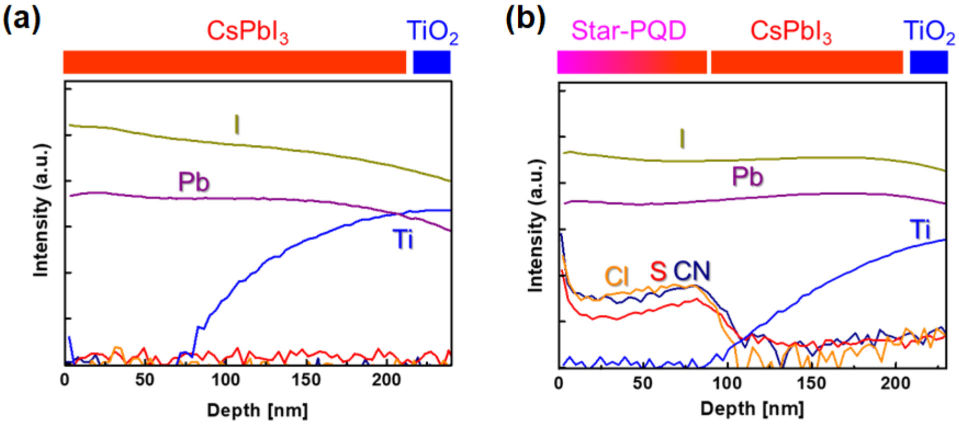

Depth Profile Analysis

Cs+ Gun, Impact Energy: 5keV, Current: 5nA, Raster Size: 300㎛×300㎛, Analysis Area: 60um(Φ),

Detected Ion: 133Cs12C14N, 133Cs32S, 133Cs35Cl, 133CS48Ti, 133Cs127I, 133Cs208Pb

Fig. S11 Time-of-flight secondary ion mass spectrometry(ToF-SIMS) depth profiles of Pb, I, Ti, S, Cl, and CN in (**a**) bare-PQD and (**b**) Star-PQD hybrid films

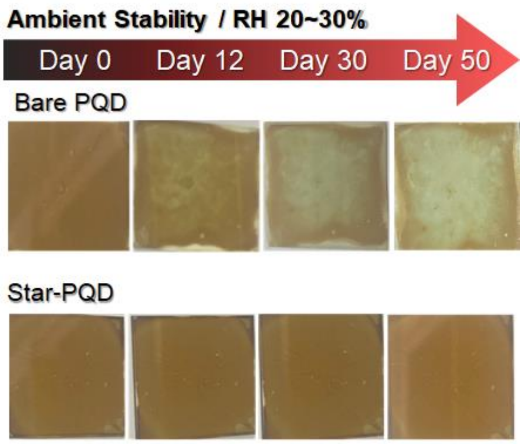

Fig. S12 Glass/bare PQD and glass/Star-PQD film images before and after aging for 50 days stored in ambient conditions (20–30% RH)

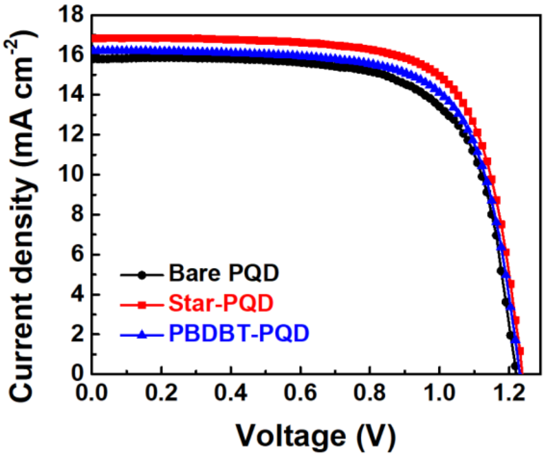

Fig. S13 Current–voltage (*J*–*V*) curves of the bare PQD, Star-PQD and PBDBT-PQD under AM 1.5G illumination

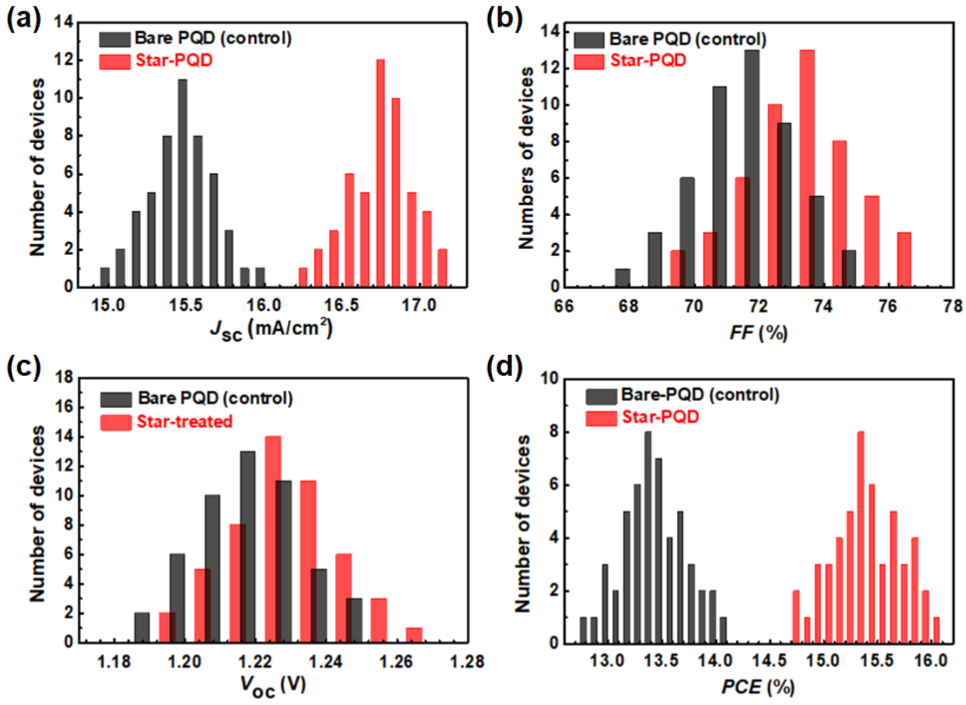

Fig. S14 Device histograms of Star-PQD- and bare PQD (control)-based solar cells: (**a**) J_{SC}, (**b**) FF, (**c**) V_{OC}, and (**d**) PCE, respectively

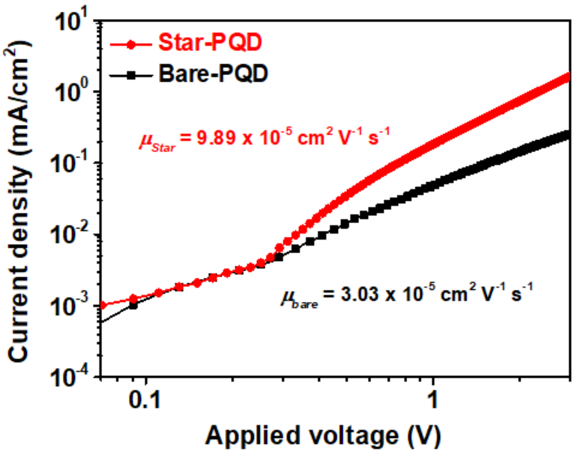

Fig. S15 SCLC fitting hole mobility results obtained from dark J–V measurements of hole-only devices compromising Star-PQD and bare PQD

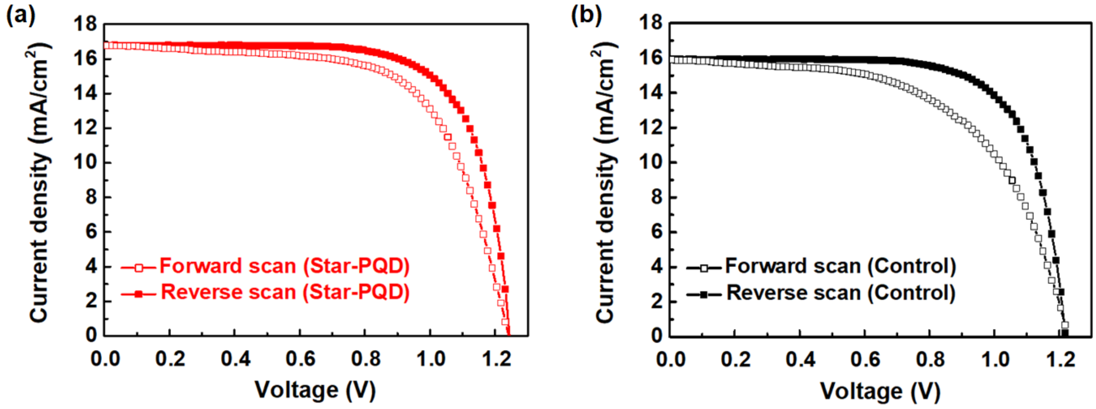

Fig. S16 Device hysteresis curves of the (**a**) Star-PQD- and (**b**) bare PQD (control)-based solar cells obtained from the *J*–*V* measurements with forward and reverse scans

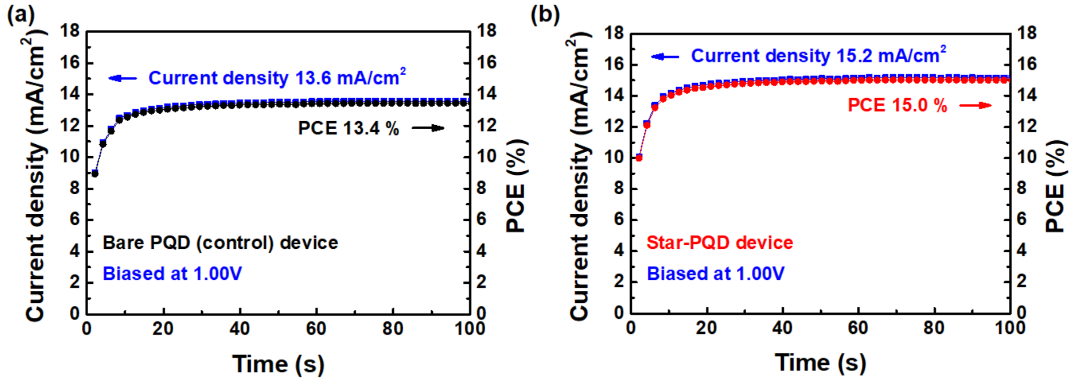

Fig. S17 SPO measurements of the (**a**) bare PQD (control)- and (**b**) Star-PQD-based solar cells at the maximum power point of 1.00 V for 100s

Table S1 Adsorption energy according to the adsorption element of Star-TrCN

Adsorption element	PbI_2 termination		CsI termination	
	PbI_2 [eV]	I vacancy [eV]	CsI [eV]	I vacancy [eV]
-Cl	0.49	0.95	0.03	-0.42
-CN	0.12	0.31	0.03	-0.72
-CO	0.06	0.47	-0.15	-0.30

Table S2 TRPL spectra parameters of bare and Star-PQD film

	**A_1 (%)**	**τ_1 (ns)**	**A_2 (%)**	**τ_2 (ns)**	**τ_{avg} (ns)**
Bare PQD	53.6	2.41	46.4	12.20	19.6
Star-PQD	66.2	1.95	33.8	8.86	8.0

The carrier lifetime (τ_{avg}) was calculated by the follwing formula:

$$\tau_{avg} = (A_1\tau_1^2 + A_2\tau_2^2)/(A_1\tau_1 + A_2\tau_2)$$

Table S3 Summary of device performance and stability of $CsPbI_3$-PQD and Hybrid $CsPbI_3$-PQD (HPQDs) solar cells at different test condition

**PQD type**	**PCE [%]**	**Test condition**		**Retention [%]**	**Refs.**
		**RH [%]**	**Duration**		
$CsPbI_3$	14.1	0 (N_2 atmosphere)	168 h	87	*Adv. Energy Mater.*, **2019**, *9*, 1900721
$CsPbI_3$	15.2	0 (N_2 atmosphere)	66 h	84	*Adv. Mater.* **2020**, *32*, 2001906
$CsPbI_3$	14.9	0 (N_2 atmosphere)	130 h	90	*Adv. Mater.* **2020**, *32*, 2000449
$CsPbI_3$	14.3	10	100 h	90	*J. Am. Chem. Soc.* **2020**, *8*, 3775
$CsPbI_3$	14.6	0 (N_2 atmosphere)	325 h	80	*Angew. Chem., Int. Ed.* **2020**, *59*, 22230
$CsPbI_3$	14.2	20-30	720 h	60	*ACS Energy Lett.* **2020**, 5, 3322
$CsPbI_3$	15.3	20-30	288 h	90	*ACS Energy Lett.* **2021**, *6*, 2229
$CsPbI_3$	15.3	20-30	90 h	80	*Adv. Mater.* **2021**, 2105977
HPQD (Graphene)	11.6	0 (N_2)	720 h	90	*Adv. Energy Mater.* **2018**, *8*, 1800007
HPQD (ITIC)	12.7	-	-	-	*Adv. Mater.* **2019**, *31*, 1900111
HPQD (PBDB-T)	13.8	20-30	350 h	63	*J. Mater. Chem. A*, **2020**, *8*, 8104–8112
HPQD (PCBM)	15.1	0 (N_2)	336 h	70	*Nat. Commun.*, **2021**, *12*, 466
HPQD (Y6-F)	15.0	20-30	140 h	70	*Adv. Funct. Mater.* **2021**, *31*, 2101272
HPQD (F6TCNNQ)	15.3	20-30	90 h	80	*Adv. Mater.* **2021**, *34*, 2105977
**HPQD (Star-TrCN)**	**16.0**	**20-30**	**400 h**	**91**	**This work**
			**1000 h**	**72**	**This work**

RH: relative humidity and room temperature

Supplementary References

[S1]J. Kim, B. Koo, W.H. Kim, J. Choi, C. Choi et al., Alkali acetate-assisted enhanced electronic coupling in $CsPbI_3$ perovskite quantum dot solids for improved photovoltaics. Nano Energy **66**, 104130 (2019). https://doi.org/10.1016/j.nanoen.2019.104130

[S2]J. Kim, S. Cho, F. Dinic, J. Choi, C. Choi et al., Hydrophobic stabilizer-anchored fully inorganic perovskite quantum dots enhance moisture resistance and photovoltaic performance. Nano Energy **75**, 104985 (2020). https://doi.org/10.1016/j.nanoen.2020.104985

[S3]S. Lim, J. Kim, J.Y. Park, J. Min, S. Yun et al., Suppressed degradation and enhanced performance of $CsPbI_3$ perovskite quantum dot solar cells via engineering of electron transport layers. ACS Appl. Mater. Interfaces **13**(5), 6119–6129 (2021). https://doi.org/10.1021/acsami.0c15484
